# Occupational therapy interventions for functional neurological disorder: a scoping review

**DOI:** 10.3389/fneur.2026.1926640

**Published:** 2026-10-30

**Authors:** Clare Nicholson, Fabiana Lorencatto, Mahinda Yogarajah, Glenn Nielsen

**Affiliations:** 1 The National Hospital for Neurology and Neurosurgery, University College London Hospitals NHS Foundation Trust, London, United Kingdom; 2 Centre for Behaviour Change, University College London, London, United Kingdom; 3 Department of Clinical and Experimental Epilepsy, Institute of Neurology, University College London, London, United Kingdom; 4 Neuroscience Research Institute, City St George's University of London, London, United Kingdom

**Keywords:** activities of daily living (ADL), Functional Neurological Disorder (FND), multidisciplinary rehabilitation, occupational engagement, occupational performance, occupational therapy, rehabilitation

## Abstract

**Introduction:**

Functional Neurological Disorder (FND) is a common and disabling condition for which multidisciplinary rehabilitation is recommended; however, the specific contribution of occupational therapy remains poorly defined. This scoping review aimed to map and characterize occupational therapy interventions for people with FND, examine how these are delivered and evaluated within rehabilitation and identify priorities for future research.

**Methods:**

A scoping review was conducted using Joanna Briggs Institute (JBI) methodology. Peer-reviewed English-language intervention studies involving adults (≥18 years) with FND who received occupational therapy delivered independently or as part of multidisciplinary rehabilitation and reporting treatment outcomes were included. Descriptive analysis was undertaken. OVID Medline, Embase, APA PsycInfo and CINAHL Plus were searched from inception to July 2026, supplemented by reference list screening.

**Results:**

Forty-one studies (*n*= 1,284 participants; sample sizes 1–201) were included, with only one randomized study. Occupational therapy was predominantly delivered within multidisciplinary rehabilitation; only one study included a subgroup receiving occupational therapy alone. Despite considerable variation in intervention reporting, common occupational therapy intervention components included facilitating occupational engagement, movement retraining and anxiety management, with symptom management strategies integrated into functional activities to promote confidence and independence. Improvements in symptom severity, functional ability, mental health, and quality of life were reported following multidisciplinary rehabilitation including occupational therapy. However, findings were primarily derived from uncontrolled studies, and interpretation was further limited by heterogeneity in intervention approaches, inconsistent use of outcome measures, and variable follow-up.

**Discussion:**

Although occupational therapy was included in all eligible studies, occupational therapy-specific interventions and associated outcomes were inconsistently described and evaluated, limiting understanding of occupational therapy's distinct contribution within multidisciplinary rehabilitation. Future research should focus on clearly defining and evaluating the components of occupational therapy interventions.

## Introduction

Functional Neurological Disorder (FND) is characterized by neurological symptoms that arise from a problem with how the nervous system functions, rather than from a structural or identifiable neurological disease (1). Symptoms, which can include weakness, tremor, cognitive difficulties, and functional seizures, are genuine, involuntary, and can impact every facet of daily life, causing distress and disability (2). Fatigue and pain are also commonly experienced alongside the symptoms of FND and can adversely affect function and quality of life (3). Diagnosis is based on identification of positive clinical signs and internal inconsistency on neurological examination (1). Functional Neurological Disorder is best understood within a biopsychosocial framework; recognizing the interplay between biological, psychological and social factors in the development and persistence of symptoms. Its classification across neurological and psychiatric diagnostic frameworks reflects its position at the interface of these disciplines (4, 5). Historically, a range of diagnostic terms have been used to describe FND, including conversion disorder, psychogenic, non-organic and medically unexplained neurological symptoms. Over time, terminology has evolved alongside changes in understanding and classification of the disorder, with Functional Neurological Disorder (FND) now the preferred term within clinical practice and research (4, 5). FND is one of the most common conditions encountered in neurology services, accounting for approximately 5%−15% of presentations, and predominantly affects adults of working age, with a higher prevalence in women than men (6).

Occupational therapy is recognized as an integral component of multidisciplinary rehabilitation for FND (7). Occupational therapy practice is guided by conceptual models that emphasize understanding the person in the context of their environment, highlighting the inter-relationship between physical and mental health (8, 9). These models recognize occupational performance as a person's ability to choose and satisfactorily perform meaningful occupations. Occupational performance arises from the dynamic interaction between the person, their occupations (daily activities), and the environment, and is influenced by physical, cognitive, emotional and social factors (10, 11). Occupational therapy assessment focuses on identifying how these interacting factors affect occupational performance and participation, with individually tailored rehabilitation strategies used to reduce identified barriers and develop self-management skills. This occupation centered perspective complements the contributions of other rehabilitation professionals by addressing the impact of symptoms on everyday functioning and enabling people to re-engage in valued activities and life roles (12).

Over the past decade, interest in rehabilitation for FND has grown substantially, reflected in an expanding body of research and profession specific clinical guidance. Existing reviews have examined rehabilitation from several perspectives, including specific symptom presentations such as functional motor symptoms (13, 14), the outcomes of interventions evaluated in clinical trials (15), the influence of symptom chronicity on rehabilitation outcomes (16), and rehabilitation delivered within specific settings, including inpatient (17), and outpatient services (18). Together, these reviews have advanced understanding of rehabilitation for FND but have examined rehabilitation through specific clinical, methodological or service-based lenses.

Gardiner et al. highlighted occupational therapy as an important component of multidisciplinary rehabilitation for FND. Recognizing the limited occupational therapy-specific evidence available, they proposed a research agenda for the profession (19). Since then, the occupational therapy literature has expanded, with publications describing occupational therapy specific interventions and clinical approaches, alongside the development of expert consensus recommendations for occupational therapy practice in FND (19–28). These publications have strengthened the evidence base and informed clinical practice. Nevertheless, important knowledge gaps remain regarding the occupational therapy interventions evaluated in published intervention studies, their delivery across rehabilitation settings, and how outcomes have been measured and reported.

The primary aim of this scoping review was to map and characterize occupational therapy interventions reported within the FND rehabilitation literature. The secondary aims were to describe how these interventions were delivered, examine how outcomes have been measured and reported, and identify knowledge gaps to inform future research and clinical practice. The following questions were investigated:

- What interventions are used by occupational therapists in the treatment of FND?
- How are these interventions delivered?
- How are the outcomes of occupational therapy interventions measured?
- What clinical outcomes have been reported following rehabilitation that includes occupational therapy for FND?

## Methods

### Study design

This scoping review was conducted to map and characterize occupational therapy interventions for FND reported in peer-reviewed intervention studies. A scoping review methodology was considered appropriate given the broad nature of the review questions and the aim of mapping the range and characteristics of occupational therapy interventions reported in this emerging area of practice. The review followed Joanna Briggs Institute (JBI) methodology for scoping reviews, aiming to describe the nature of the available evidence, identify knowledge gaps, and inform future research rather than evaluate intervention effectiveness (29). It was reported in accordance with the PRISMA extension for Scoping Reviews (PRISMA-ScR) checklist (30). The review methods, including the eligibility criteria, search strategy and data charting approach, were defined a priori.

### Inclusion criteria

Eligibility criteria were developed using the JBI population-concept-context (PCC) framework for scoping reviews (29).

### Participants

Adults (≥18 years) with a diagnosis of FND.

### Concept

Interventions delivered by occupational therapists to people with FND.

### Context

Interventions delivered by occupational therapists working independently or as part of a multidisciplinary team, in all settings (inpatients, outpatients, community).

### Types of studies

All peer-reviewed published intervention studies written in English and reporting intervention outcome data.

### Exclusion criteria

Studies were excluded if they included participants aged under 18 years, did not include an occupational therapy intervention, did not report intervention outcomes, were not primary peer-reviewed intervention studies or were published in a language other than English. Given the increasing volume of potentially eligible evidence, the review was restricted to primary studies published in peer-reviewed journals to provide a clearly defined and reproducible scope and a consistent level of peer-review and editorial oversight.

### Search strategy

A literature search was undertaken using electronic databases, OVID Medline, Embase, APA PsycINFO and CINAHL Plus (EBSCOhost); searched from inception to 28 July 2026. No date restrictions were applied to ensure comprehensive mapping of the available evidence, consistent with the objectives of the review. Historical and contemporary terminology relating to FND were incorporated into the search strategy to account for changes in diagnostic terminology over time. The search strategy was developed in consultation with a health sciences librarian at University College London (UCL) using Boolean combination of keywords and medical subject headings (MeSH). Reference lists of studies meeting the eligibility criteria, relevant systematic reviews and scoping reviews were hand searched to identify additional eligible studies.

Interventions terms were combined with condition terms using the AND search function as follows; occupational therap^*^ OR physical therap^*^ OR multidisciplinary OR interdisciplinary treatment OR program^*^; AND functional neurological disorder OR functional neurological symptom OR conversion disorder^*^ OR psychogenic OR medical^*^ unexplained OR hysteria OR dissociative disorder^*^ OR functional movement disorder^*^ OR functional motor symptom^*^. The full search strategy is provided in Supplementary material (File 1).

### Study selection

Titles and abstracts of all articles identified through the search were screened against the inclusion criteria. Full-text versions of potentially eligible studies were retrieved and assessed for final inclusion (17). Screening was conducted by CN. To verify consistency in the application of the eligibility criteria, GN independently screened a random 10% sample at each stage of the screening process (title/abstract and full-text). Any uncertainties regarding study eligibility were discussed and resolved by consensus.

### Data extraction

A data charting form was developed to extract key information from each study (Supplementary material File 3). Extracted data included author(s), year of publication, study design, participant characteristics, sample size, setting, professions involved, description of intervention, treatment intensity, outcome measures, and treatment outcomes. Study characteristics were reported as described in the original publications.

### Data analysis

Where appropriate, descriptive statistics (frequencies, proportions and weighted means where sufficient data were available) were used to summarize study characteristics and intervention features. Findings were narratively synthesized according to the review objectives.

Occupational therapy interventions were categorized using an inductive approach, with intervention components grouped according to similarities in their focus and intended therapeutic outcomes. Categories were derived directly from the included studies, rather than imposed a priori. Where intervention components were explicitly attributed to occupational therapists in the original publications, they were classified as occupational therapy specific interventions. Components not attributed to a specific profession were reported as multidisciplinary rehabilitation components. Consistent with JBI guidance for scoping reviews, formal critical appraisal of methodological quality was not undertaken.

## Results

A total of 901 records were screened, of which 41 met the inclusion criteria (published between 1985 and July 2026). Figure 1 presents the study selection process. Details of the excluded full-text articles and reasons for exclusion are provided in Supplementary Material File 2.

**Figure 1:**
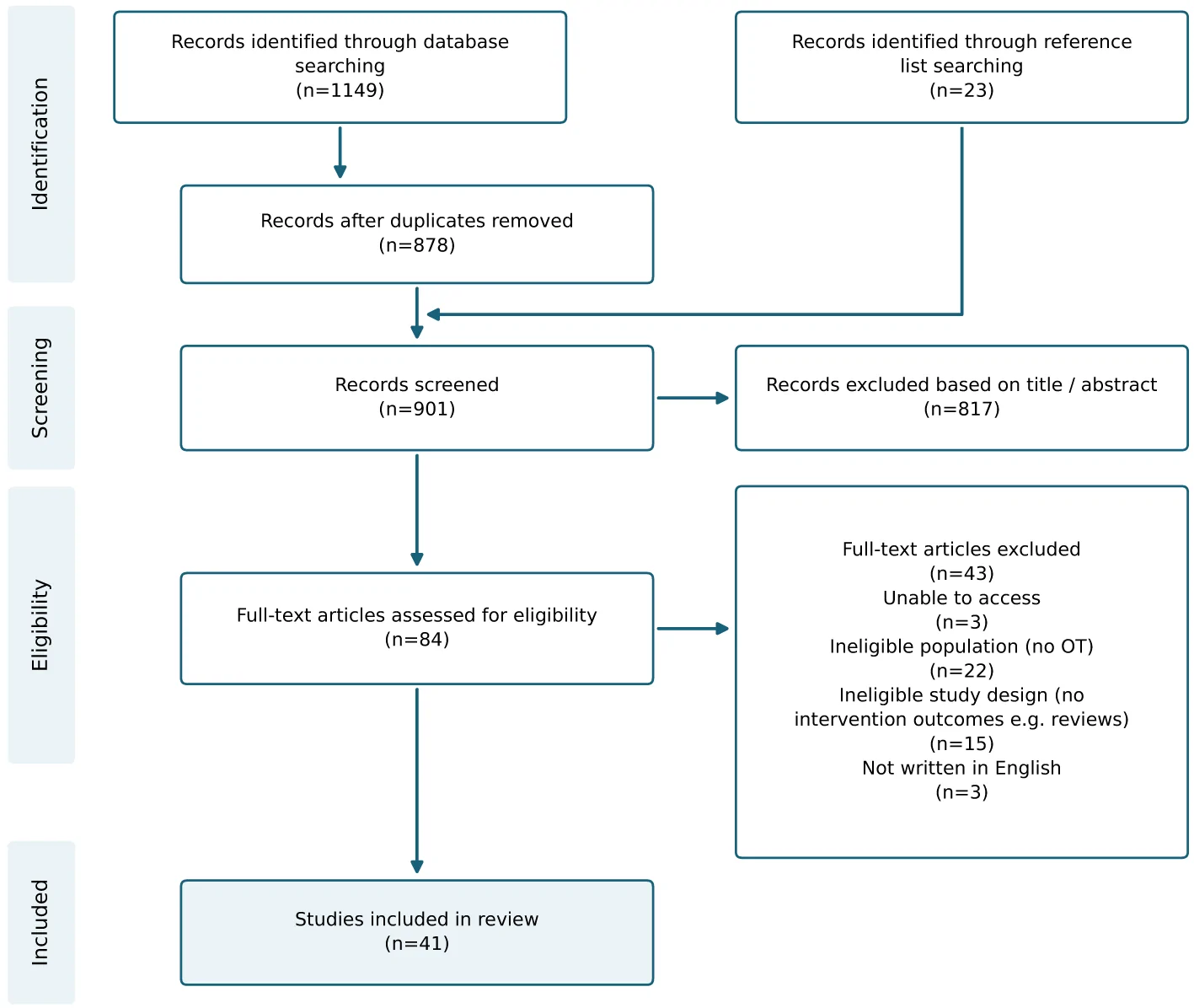
PRISMA-ScR. Flowchart of study selection and inclusion process.

An overview of the 41 included studies is presented in Table 1. The evidence base was dominated by uncontrolled observational studies, with only one randomized (waiting-list-controlled) study (31) and one retrospective cohort study with a historical control group (32). Sample sizes ranged from 1 to 201 participants. Full study summaries are provided in Supplementary material (File 3, 4).

**Table 1:** Summary characteristics of the included studies and participants (*n* = 41).

Characteristic	Summary
Study characteristics	
Publication years	1985–2026
Study design, *n*	
Randomized study with waiting-list control	1
Retrospective cohort study with historical control	1
Retrospective cohort studies	12
Prospective cohort studies	3
Case series	14
Case reports	10
Participants, *n*	
Total (range)	1,284 (1–201)
Sex and gender, *n* (%)	
Studies reporting sex	36/41
Female	893/1,274 (70.1)
Male	381/1,274 (29.9)
Non-binary	1
Age	
Weighted mean age	42.7 years (25 studies)
Reported age range	18–82 years
FND presentation studied, *n*	
Mixed functional neurological symptoms	18
Functional motor symptoms	14
Functional weakness/paralysis	7
Functional seizures	1
Persistent postural perceptual dizziness (PPPD)	1
Symptom duration	
Studies reporting symptom duration	33/41
Weighted mean (range)	4.9 years (1 day−40 years)^1^
Treatment setting, *n*	
Inpatient	29
Outpatient	10
Community	1
Mixed inpatient/outpatient	1

^1^Weighted mean calculated from studies with sufficient data for pooled analysis (*n* = 13).

Mixed functional neurological symptoms includes one cohort with predominant pain.

## Population characteristics

The 41 studies comprised 1,284 participants, with a predominance of women and a weighted mean age of 42.7 years across the 25 studies reporting mean age. Mixed functional neurological symptoms and functional motor symptoms were the most frequently studied presentations. Symptom duration varied substantially, ranging from 1 day to 40 years among studies reporting these data (Table 1).

## Intervention delivery

Occupational therapy was delivered within multidisciplinary rehabilitation or alongside other therapies in all 41 studies, although a subgroup of 11 participants in one study received occupational therapy alone (27). Rehabilitation was most commonly delivered in inpatient settings (Table 1), with treatment taking place in person in all studies but one, which provided a combination of in-person and tele-health sessions (27). Table 2 summarizes the setting, treatment duration and intensity, occupational therapy assessment tools and interventions reported in the studies.

**Table 2:** Characteristics of occupational therapy interventions across included studies (*n* = 41).

Reference	Primary FND presentation	*n*	Setting & duration	OT intensity	Occupational therapy assessment tools and intervention
Randomized study with waiting-list control					
Jordbru et al. (31)	Motor	60	Inpatient 3 weeks	Not specified	MDT. Assessment tools not specified. Adaptive physical activity within a CBT framework. OT intervention not specifically described.
Retrospective cohort study with historical control					
Czarnecki et al. (32)	Motor	60	Outpatient 5 days	Twice daily PT & OT for 5 days	MDT. Assessment tools not specified. Education. Motor retraining. OT intervention not specifically described.
Prospective cohort studies					
Rush et al. (46)	Motor	45	Outpatient 5 days	3–4 1 hour sessions over 5 days	MDT. COPM. Motor re-training, relaxation exercises, introduction of cognitive and environmental challenges during functional tasks.
Schmidt et al. (59)	Motor	31	Inpatient Range 10–35 days	Not specified	MDT. Assessment tools not specified. Physical therapies: motor re-training, enhancement of body awareness. OT intervention not specifically described.
Demartini et al. (54)	Motor, seizures, sensory	66	Inpatient 4 weeks	Therapy delivered Monday–Friday Not specified	MDT. COPM. Combined cognitive and physical rehabilitation. OT intervention not specifically described.
Retrospective cohort studies					
Foong et al. (63)	Mixed symptoms + 51 predominant pain	100	Inpatient 4 weeks	Not specified	MDT. COPM. OT intervention not described.
Le Perf et al. (66)	PPPD, dizziness	20	Inpatient 4 weeks	Not specified Treatment delivered 5 days per week for 4 weeks.	MDT. Assessment tools not specified. Balance group and functional mobility training.
Schneider et al. (50)	Motor, seizures	30	Inpatient Mean, 4 weeks	1 daily session of OT for 30–45 min, 5 days per week.	MDT. Assessment tools not specified. OT intervention not described.
Saunders et al. (40)	Motor, seizures, cognitive, mixed symptoms, fatigue	52	Inpatient Range 3–76 weeks	Not specified	MDT. Assessment tools not specified. Activity planning, relaxation strategies, building confidence, and life skills.
Polich et al. (48)	Motor, seizures	22	Inpatient 2 weeks	3+ h delivered daily over 5–6 days per week.	MDT. (PT and OT measures); IRF-PAI. Aquatic therapy co-delivered by PT and OT. Therapeutic recreation sessions. OT consensus recommendations for FND applied. Self-management plan.
McCombs et al. (27)	Motor, seizures, speech	77	Outpatient 1–6 months	1-h OT, starting with once weekly–once every few weeks.	OT (11)/MDT (66). 11 patients stand-alone OT, 45 patients' concurrent psychotherapy, 39 concurrent PT, 17 concurrent SLT. ^**^AASP. 8 OT sensory-based treatment modules (26). In person and remote.
Callister et al. (43)	Motor	201	Outpatient 5 days	3–4 1-h sessions daily between PT & OT.	MDT. COPM. Therapeutic screening. Motor retraining, relaxation exercises, self-management strategies. Functional task practice.
Glass et al. (56)	Motor, seizures	100	Outpatient Up to 6 sessions	Baseline evaluation & up to 6 outpatient visits.	MDT. Assessment tools not specified. Education, otherwise not described.
Jacob et al. (33)	Motor	32	Inpatient 5 days	3 h per day of physical therapies: PT, OT, SLT	MDT. Assessment tools not specified. OT intervention not specifically described. Physical therapies: motor retraining, relaxation strategies. Assistive devices removed as able. Mental practice (imagery) retraining.
Dahlhauser et al. (20)	Motor	36	Outpatient 5 days	Up to 2, 45-min sessions of OT & PT daily for 1 week (up to 10 sessions of OT).	MDT. COPM. Goal directed. Functional task practice with divided attention, relaxation techniques.
McCormack et al. (38)	Motor	33	Inpatient Median 101 days	Not specified	MDT. Clinician-rated ADL independence (admission/discharge): largely independent, somewhat dependent, or mostly/fully dependent. Standardized OT specific tools not reported. Psychoeducation. Assess baseline ADLs and safety, role and routine, home and social environments and impact on function. Graded task practice. motor retraining, relapse prevention.
Saifee et al. (53)	Motor, fatigue	26	Inpatient Median 24 days, range 15–32 days	Monday–Friday Intensity not specified	MDT. Assessment tools not specified. Combined cognitive and physical rehabilitation. OT intervention not specifically described.
Case series					
Gilmour et al. (45)	Motor	20	Outpatient 5 days within a 1-year pathway	1-h OT delivered on day 2 & 3 of the 5-day programme.	MDT. COPM. Goal-directed task specific rehabilitation otherwise not described.
Geary et al. (49)	Motor, weakness, dizziness, seizures.	3	Inpatient 13–18 days	3 h of therapy per week between PT, OT.	MDT. Assessment tools not specified. Education re: FND. OT intervention otherwise not described.
Reid et al. (44)	Motor	18	Outpatient 5 days	90 min daily	MDT. COPM. Autonomic regulation using relaxation therapies and motor retraining. Functional task practice, with mental imagery.
Hebert et al. (58)	Motor	17	Inpatient Up to 14 days (median 7.5 days)	Not specified	MDT. Assessment tools not specified. Physical therapies: motor retraining, work on symptom triggers, maintaining factors. OT intervention not specifically described.
Faul et al. (34)	Motor	14	Inpatient 5 days	3 h daily of physical therapy btw PT, OT, SLT	MDT. Assessment tools not specified. *MORE* treatment protocol: motor retraining programme.
Petrochilos et al. (47)	Motor, seizures, sensory, cognitive, mixed symptoms	78	Outpatient 5 weeks	2, 1-hour sessions of OT per week for 5 weeks & MDT review	MDT. COPM. Group FND education, graded goal setting, graded activity practice, fatigue, pain, anxiety, symptom management, routine development, community access, vocational rehabilitation, relapse prevention.
Jimenez et al. (64)	Motor, seizures, mixed symptoms	49	Inpatient 3–4 weeks	Treatment delivered Monday–Friday OT Intensity not specified.	MDT. Assessment tools not specified. Education, biofeedback/relaxation techniques
Richardson et al. (35)	Motor, seizures, mixed symptoms	12: IP (11), OP (1)	Inpatient : 5 days. Outpatient: 3 hours total	Inpatient: 2–4 h of therapy daily for 5 days. Outpatient: 3 h total input.	MDT. Assessment tools not specified. Functional task practice. Graded exposure.
Heruti et al. (68)	Motor	34	Inpatient Not specified	Not specified	MDT. Assessment tools not specified. OT intervention not described
Speed et al. (52)	Motor	10	Inpatient Mean 11.8 days	Not specified	MDT. Assessment tools not specified. Positive reinforcement of normal function, functional standing practice.
Vatine et al. (61)	Functional paralysis/weakness	2	Inpatient 4–6 weeks	Not specified	MDT. Assessment tools not specified. Functional task practice, motor retraining targeting the upper limb.
McDade et al. (41)	Seizures	16	Inpatient 3–6 months	Not specified	MDT. Assessment tools not specified. OT intervention not described
Fishbain et al. (37)	Functional paralysis	4	Inpatient 2 weeks−3.5 months.	Not specified	MDT. Assessment tools not specified. Education, EMG feedback.
Withrington & Parry (36)	Weakness	5	Inpatient 4 weeks−6 months	Not specified	MDT. Assessment tools not specified OT intervention not described
Case reports					
Zheng et al. (51)	Seizures, tremor, weakness, speech, cognitive	1	Inpatient 8 days	Daily	MDT. Assessment tools not specified. Graded activity practice incorporating using CBT informed strategies.
Ashrafzadeh et al. (65)	Motor, functional seizures with Cushing's Disease.	1	Inpatient Not specified	Not specified	MDT. Assessment tools not specified. OT for hand dexterity: writing, drawing, stress ball.
Hebb et al. (60)	Motor	1	Inpatient 8 days	Not described	MDT. Assessment tools not specified. OT intervention not specifically described.
MacLean et al. (25)	Mixed symptoms	2	Outpatient CS 1: 8 weeks CS 2: 8 weeks	CS1: OT once every 2 weeks for 8 weeks (7 sessions). CS2: OT once weekly for 8 weeks (7 sessions).	MDT. AASP, COPM. Physical and functional + sensory processing assessment, review of daily routines and occupational performance; individualized sensory-based treatment plan.
Henry et al. (39)	Seizures, weakness	1	Inpatient 12 months	Not described	MDT. Assessment tools not specified. OT intervention not described
Joos & Halmer (57)	Motor	1	Inpatient 8 weeks	Not specified	MDT. Assessment tools not specified. Manual therapy to relax muscles alongside mirror therapy.
Hardin & Carson (62)	Mixed symptoms	1	Inpatient 22 days	Not specified	MDT. Assessment tools not specified. PT and OT –motor-retraining, gradual reduction of aids.
Yam & Rickards (55)	Mixed symptoms	1	Inpatient 3 months	Not specified	MDT. Assessment tools not specified. Psychoeducation; motor retraining; attention and communication training; graded exposure; IADL retraining; anxiety management and relaxation; community reintegration.
Chou et al. (67)	Weakness	1	Inpatient Not specified	Not specified	MDT. Assessment tools not specified. OT intervention not specified.
Paget & Rigby (42)	TBI, spinal injuries and functional overlay.	1	Community 10 days post injury for 4 months + 9 months post injury (after self-referral) for 3 months.	Not specified	MDT. AMPS, RBMT, COTNAB. Cognitive and vocational rehab, social activities.

PT, physiotherapy, OT, occupational therapy; SLT, speech and language therapy; MDT, multidisciplinary team; ADLs, activities of daily living; IADL, instrumental activities of daily living; UL, upper limb.

Outcome measures: COPM, Canadian occupational performance measure; AASP, adolescent/adult sensory profile; AMPS, assessment of motor and process skills; RBMT, the rivermead behavioral memory test; COTNAB, The Chessington OT neurological assessment battery; IRF-PAI, inpatient rehabilitation facility patient assessment instrument.

^**^Details regarding concurrent therapies were clarified through communications with the study authors.

Treatment duration varied widely. Inpatient rehabilitation ranged from 5 days to 12 months, with short five-day intensive programmes predominantly offered to patients with motor symptoms (33–35). Longer admissions of 3 months or more were typically reported in cohorts with mixed functional symptoms, psychiatric comorbidities, or complex presentations (36–41, 55). The single community-based study reported 7 months of treatment, delivered across two separate intervention periods within a 12-month period (42).

Outpatient treatment duration varied considerably, ranging from 3 h of total input (35) to 6 months (27). Six studies reported intensive five-day programmes for motor symptoms (20, 32, 43–46) while other programmes lasted 5–12 weeks (25, 47) for mixed functional symptoms.

Treatment intensity was inconsistently reported and varied considerably according to setting and intervention model. Five-day intensive inpatient programmes typically involved daily multidisciplinary therapy (33–35). Longer inpatient admissions (i.e., 2–4 weeks) provided between 3 h of therapy per day and 3 h per discipline (including occupational therapy) per week (48–51). Where reported, outpatient occupational therapy ranged from one to two sessions daily within intensive five-day programmes, to three to four 60-min sessions per week. Programmes that extended over several weeks or months, often began with higher intensity and tapered over time (25, 27).

Planned follow-up after treatment was reported in 25 studies and most commonly occurred at six and 12 months (25, 31–33, 35–37, 39, 41, 43, 45, 47, 49, 50, 52–61).

The composition of multidisciplinary teams varied across studies but most commonly included physiotherapy (*n* = 40) and psychological therapy (*n* = 35), followed by psychiatry/neuropsychiatry (*n* = 19), speech and language therapy (*n* = 16), rehabilitation medicine / physiatry (*n* = 16) and neurology (*n* = 14). Further study-level details of multidisciplinary team composition are provided in Supplementary Files 3, 4 (Tables 1, 2). Despite the prominence of multidisciplinary rehabilitation, explicit interdisciplinary co-delivery involving occupational therapists was described in only three studies. Occupational therapists were involved in joint treatment with physiotherapists, including task specific practice, aquatic therapy, and therapeutic recreation, and worked together with psychological therapies to support the transfer of symptom management strategies across different contexts (47, 48, 62).

## Intervention components

To distinguish occupational therapy from broader multidisciplinary rehabilitation, intervention components were categorized according to whether they were explicitly attributed to occupational therapists in the original study reports. Where the contribution of occupational therapy could not be distinguished from that of the wider multidisciplinary team, intervention components were reported as multidisciplinary rehabilitation components.

A range of intervention components were described across the included studies.

## Multidisciplinary rehabilitation components

### Goal setting

Goal setting was reported in 20 studies (20, 25, 27, 31, 33, 40, 43, 45–47, 49, 51–55, 59, 60, 62, 63). Descriptions rarely specified the process of goal setting, for example whether patients were involved and which professions contributed.

## FND education as part of treatment

Education about FND as a rehabilitation tool was reported in 21 studies (31, 32, 35–38, 40, 41, 43, 45, 47–52, 55, 56, 64–66). It was delivered individually or within a group, with one study including significant others (47). Education content and the professionals involved in delivery were frequently unspecified. Table 3 summarizes educational components reported in the literature.

**Table 3:** Summary of education components used in FND rehabilitation.

Education component	Description
Developing a therapeutic relationship.	Building rapport and validating symptoms as genuine and disabling prior to providing education (34, 35, 47).
Pathophysiological explanations of FND	The nervous system is structurally intact and undamaged. Symptoms arise due to a “disconnect,” “misfiring,” or “block in transmission” between the brain and the body (31, 32, 45, 46, 50, 68). Hardware/software analogy: Intact brain structure (“hardware”) with disrupted functional processing (“software”) (68).
Rule-in signs and exclusion of disease	Explanation of how positive ‘rule -in' signs and normal diagnostic investigations are used in making the diagnosis of FND (45–47, 49, 57).
Attention and symptom modulation	Explanation of how inadvertent self-focused attention can exacerbate symptoms and redirection of attention can reduce them (36, 47, 50).
Psychoeducation	Education and discussions re: interrelationship between mental health and physical symptoms. Exploration of symptom triggers, warning signs and unhelpful thoughts and beliefs e.g., challenging the belief that the body was “damaged” (34, 39, 40, 42, 49, 50).
Biopsychosocial formulation	Collaborative formulation to support understanding of the interaction between biological, psychological and social factors in symptom development and maintenance (31, 33, 37, 47, 49, 50, 67).
Symptom reversibility	Emphasis that function is potentially recoverable due to the absence of structural neurological damage (31, 32, 36, 45, 46, 54, 57).

## Occupational therapy specific interventions components

Descriptions of occupational therapy were provided in 23 studies, although the level of detail varied considerably. Occupational therapy assessment tools and interventions across the included studies are summarized in Table 2. Intervention components were grouped based on their focus and intended therapeutic outcomes, yielding seven treatment categories: (i) facilitating occupational engagement; (ii) movement retraining; (iii) anxiety management and relaxation strategies; (iv) sensory modulation interventions; (v) community re-integration; (vi) care, aids and adaptations; and (vii) relapse prevention and self-management planning.

## Facilitating occupational engagement

Facilitating occupational engagement was described in 15 studies (25, 27, 35, 38, 40, 42–44, 46, 47, 51, 52, 55, 61, 65). These interventions focused on identifying barriers to participation in daily activities and roles and supporting graded re-engagement through tailored activity planning, routine development, and strategies to promote independence and confidence. Graded exposure to daily activities was also described (25, 27, 35, 38, 40, 46, 47, 51, 55). This approach incorporated pacing strategies to manage pain and fatigue, with a focus on the gradual re-introduction of previously avoided activities to enhance participation while minimizing excessive symptom exacerbation. Graded exposure also encompassed a scaffolded approach whereby increasingly challenging tasks were introduced alongside physical symptom management strategies and techniques to address psychological barriers such as anxiety or reduced confidence. This supported patients to re-engage with feared or avoided activities.

## Movement retraining

Movement retraining was described as part of the occupational therapy role for individuals with motor symptoms. This involved teaching techniques that allowed access to normal movement, for example, strategies to divert attention away from symptoms and toward the environment or the goal of the task (32–34, 38, 43, 44, 46–48, 58, 62). Tremor entrainment or the use of rhythm to elicit change in tremor frequency and amplitude, was also used (31, 52, 58). Normal movement strategies were often combined with psychoeducation and relaxation techniques (20, 33, 46, 47, 59). As symptom control improved, patients were guided to apply these strategies within functional tasks (e.g., meal preparation, personal care, sports/leisure activities), with opportunities for both supported and independent practice (31, 38, 43, 44, 47, 52, 55, 61).

Adjuncts to movement retraining included mental imagery of normal movement and mirror therapy (33, 44, 57).

## Anxiety management and relaxation strategies

Psychoeducation was integrated into occupational therapy sessions alongside techniques targeting anxiety, including diaphragmatic breathing (20, 33, 43, 44, 46, 55), progressive muscle relaxation (20, 25, 43) and body scanning to promote mind-body awareness (55). These strategies were applied during functional tasks to reduce the impact of anxiety and hyperarousal on symptoms and function (25, 40, 47). Less frequently reported approaches included relaxation-focused biofeedback interventions, including heart rate variability biofeedback (55, 64), vagal nerve stimulation techniques, sensory-enhanced yoga, smartphone-based relaxation apps, meditation and mindfulness training (25, 44).

## Sensory modulation interventions

Two studies described occupational therapy specific sensory-based assessment and interventions for individuals experiencing sensory processing difficulties (typically hypersensitivity to light, noise, touch and movement) (25, 27). Interventions aimed to promote self-regulation of sensory symptoms by improving awareness of responses to sensory stimuli and how these may be linked with FND symptoms. Personalized symptom management plans were developed, including graded exposure to avoided stimuli alongside techniques to regulate anxiety and hyperarousal (e.g., diaphragmatic breathing). Patients were supported to integrate these strategies into daily routines to improve sensory tolerance and activity participation.

## Community reintegration

Community reintegration in the form of home visits, improving independence and confidence with accessing the community, returning to driving, voluntary work and supporting patients to stay in work and education (vocational rehabilitation) was reported in three studies (38, 47, 55).

## Care, aids and adaptations

A graded approach to the reduction of carer support and use of assistive aids was outlined in three studies (33, 47, 62).

## Relapse prevention and self-management plans

Relapse prevention and self-management planning were typically described as multidisciplinary treatment components, with occupational therapy contributing as part of the wider team. These were reported in seven studies (20, 38, 45, 47, 48, 62, 66). Plans were commonly described as a supporting tool to conclude treatment and to aid independent carryover of rehabilitation strategies post-discharge. A summary of key components of relapse prevention and self-management planning reported in the literature is provided in Box 1.

Box 1 **Key components of relapse prevention and self-management planning.**

• Written summary of the individual's biopsychosocial formulation
• Summary of known symptom triggers
• Summary of symptom warning signs of symptom escalation (e.g. emotions, feelings or physical sensations experienced prior to a symptom emerging or worsening).
• Guidance on independent use of strategies to help regulate emotions, thoughts and symptoms.
• Structured home programme to support independent practice (e.g. exercise programme, normal movement videos).
• Guidance on how to integrate rehabilitation strategies into daily activities.
• Written summary of personal learning from rehabilitation.
• Graded goals set at discharge with planned review.
• Multidisciplinary discharge meeting with the patient and significant others, providing a space for reflection on changes in symptoms, function, mood and goals.
• Guidance for significant others on ways to support the patient's independence.

## Outcome measures

More than 60 different outcome measures were used to evaluate multidisciplinary rehabilitation across physical, functional and psychological domains, although relatively few were specific to occupational therapy.

Four occupational therapy specific outcome measures were identified. The Canadian Occupational Performance Measure (COPM) was the most frequently reported (*n* = 8) (20, 25, 43, 45–47, 54, 63), followed by the adult adolescent sensory processing tool (AASP) (*n* = 2) (25, 27). The assessment of motor and process skills (AMPS) and Chessington occupational therapy neurological assessment battery (COTNAB) were each reported in one study (42).

Among the more commonly reported standardized measures used in multidisciplinary rehabilitation were the Clinical Global Impression scale for symptom severity (*n* = 6) (33, 34, 40, 47, 58, 59), Berg Balance Scale for physical functioning (*n* = 6) (40, 44, 49, 58, 60, 62), Functional Independence Measure for functional ability (*n* = 5) (31, 35, 50, 52, 58), Generalized Anxiety Disorder-7 (*n* = 4) (39, 47, 58, 63) and Patient Health Questionnaire-9 for psychological health (*n*= 4) (39, 47, 55, 63), and EQ-5D-5L for health-related quality of life (*n* = 4) (40, 45, 47, 63). Further details of commonly used standardized outcome measures are provided in Supplementary File 5 (Table 3), with study-level outcome measure data provided in Supplementary File 3 (Table 1).

In addition to standardized outcome measures, several studies reported non-standardized patient and clinician rated measures.

Non-standardized, patient self-reported measures included: visual analog scales of difficulty with activities of daily living, wellbeing, and perceived improvement following rehabilitation (32, 33, 53, 54); ordinal scales of change (34, 38, 43, 54); seizure frequency reported pre and post-treatment (41); and changes in employment status at follow-up compared with baseline (50, 56).

Clinician-rated non-standardized measures included ordinal ratings of symptom and functional change (27, 38, 43); changes in employment or benefit status (50, 56); use of adaptive/mobility aids (37, 38, 62); video assessment of movement quality (32, 35, 36); healthcare utilization (33, 41); muscle power (36); and documentation of symptom recurrence, new neurological symptoms, new treatments received, or new psychiatric or neurological diagnosis post-discharge (52).

## Treatment outcomes

Treatment outcomes were synthesized descriptively, with a distinction made between controlled and uncontrolled evidence. As occupational therapy was almost always delivered as part of multidisciplinary rehabilitation, the reported outcomes reflect rehabilitation programmes that included occupational therapy, rather than the independent effects of occupational therapy itself. All 41 studies reported improvements across one or more physical, psychological or quality of life domains, although the degree of improvement and the strength of evidence varied considerably. Controlled evidence from prospectively recruited participants came from only one study (31). A more detailed summary of treatment outcomes is provided in Supplementary material (Files 3, 4).

## Functional symptom and physical health outcomes

Jordbru et al. conducted the only randomized controlled trial including occupational therapy, comparing a 3-week inpatient multidisciplinary programme with a waiting-list control for 60 participants with functional gait disorder. The intervention, delivered within a “cognitive behavioral framework”, included education, adapted physical activity, and behavioral shaping strategies. Significant improvements were reported in the intervention group compared to controls on the Functional Independence Measure, Functional Mobility Scale, and the physical and mental subscales of the 12-Item Short Form Health Survey (SF-12). The control group crossed over to receive the intervention, and both groups were followed up at 12-months. Gains were sustained at 1 year follow-up (*n* = 40, 67% retention), for all measures except for the SF-12 mental subscale (31).

Across uncontrolled studies, improvements were reported in functional symptom severity, including seizure frequency, sensory impairment and weakness (20, 25, 27, 32–47, 49–58, 60, 62–65, 67, 68). Improvements were also observed in disability and function. Seven studies reported reduced reliance on mobility aids (33, 37–39, 51, 60, 62), while others described gains in balance, coordination, cognitive functioning, and communication abilities (25, 42, 51, 57, 65, 66).

Only one study specifically evaluated an occupational therapy intervention: an outpatient sensory-modulation programme. Improvements were reported at discharge in symptom severity, stress management and occupational performance, however most participants (*n* = 66/77) also received other concurrent therapies (See Table 2 for further details) (27).

## Mental health outcomes

Across inpatient and outpatient rehabilitation programmes, short-term improvements in depression, anxiety and stress were commonly reported using validated measures (25, 39, 40, 47, 54, 58, 63–65), (see Supplementary material File 3 for full details). Multi-disciplinary treatment was also reported to be associated with improvements in illness perceptions, with some patients describing their symptoms as more understandable and less distressing (40, 54). Two studies found that mental health outcomes were less durable than physical outcomes, with scores returning to baseline at 12 months, despite sustained physical gains (31, 58).

## Quality of life and social functioning outcomes

Several studies reported short-term improvements in quality of life and social functioning, reflected in positive changes on the Health of the Nation Outcome Scales (HoNOS)(47, 54), Health-Related Quality of Life assessment tool, the social life domain of the Sheehan Disability Scale (33), and the EuroQual 5D−5L (40, 45, 63). However, the extent to which these gains persisted was unclear. One study reported largely maintained HoNOS gains at 1 year but only 55% (36/66) of participants were reassessed (54), while others did not report outcomes beyond 6 months (33, 47), or did not report follow-up outcomes (40, 45, 63), limiting conclusions about long-term effects.

## Return to work

Vocational outcomes were reported infrequently and findings were mixed. Three studies reported improvements in vocational outcomes. At 12-month follow-up, Jordbru et al. reported that the “majority” of their cohort of 60, had returned to independent living and “many” had resumed employment although specific figures on return-to-work and independent living rates were not provided (31). Similarly, McDade and Brown, 1992 reported that, among 13 patients with functional seizures who completed 1-year follow-up, six had taken up further education or voluntary work, having previously been unoccupied (41). Vatine et al. reported that one patient with functional upper limb paralysis returned to military duties following a 4-week inpatient multidisciplinary rehabilitation programme (61). Conversely, two studies indicated that employment rates remained largely unchanged or declined at long-term follow-up (24–36 months), despite patients reporting ongoing benefits from treatment (50, 53).

## Receipt of disability benefits

Two studies reported outcomes related to disability benefit use. Saifee et al. found that receipt of benefits remained stable or increased following rehabilitation, despite reported improvements in symptoms and function at 2-year follow-up (53). Similarly, in their cohort of 30 patients with mixed functional symptoms, Schneider et al. observed an increase in insurance payouts, from 43.3% prior to admission to 60% at follow-up (mean 36 months) despite sustained improvements in motor function (50).

## Reduction in health care utilization

Reductions in healthcare utilization were reported following inpatient rehabilitation. Jacob et al. (*n* = 32; 84.4% followed up at 6 months) reported that inpatient treatment for functional movement disorder was associated with reductions in healthcare utilization with self-reported physician visits decreasing from a mean of 12.4 (SD = 17.9) at admission to 9.9 (SD = 14.6) 6 months post-treatment and emergency department attendances reduced from 2.3 (SD = 8.3) to 1.1 (SD = 2.2) over the same period (33).

McDade et al. reported that, at 12-month review, inpatient treatment for functional seizures was associated with reductions in seizure frequency, and fewer associated hospital admissions and GP (general practitioner) appointments (41).

## Discussion

This scoping review identified 41 studies of rehabilitation for FND, incorporating occupational therapy. By systematically mapping and characterizing intervention descriptions recurring components of occupational therapy practice were identified, including facilitating occupational engagement, movement retraining, sensory modulation, community reintegration, provision and weaning of assistive devices, relapse prevention and self-management support.

The occupational therapy role was often poorly defined. Where occupational therapy-specific details were available, the components of treatment were broadly consistent with consensus recommendations for occupational therapy for FND, and some recent studies explicitly drew on these recommendations to guide treatment (22, 43, 48).

Vocational rehabilitation was rarely described, despite being a recognized area of occupational therapy practice, and its prominence in treatment of other neurological conditions (69, 70). Several studies reported limited change in employment, care needs or receipt of benefits despite improvements in clinical outcomes (42, 50, 53). This raises questions about whether the vocational needs of people with FND are being adequately addressed within current rehabilitation models. Few studies reported whether vocational rehabilitation was offered to those in work or education. Vocational rehabilitation has shown demonstrable benefits in other neurological conditions (69, 70), and its potential contribution to FND rehabilitation remains largely unexplored.

Occupational therapy was delivered predominantly within specialist multidisciplinary inpatient and outpatient programmes. Evidence for community and generalist services was limited. Broader literature has identified barriers to delivering coordinated multidisciplinary care for FND, including limited access to psychological therapies, service fragmentation, funding constraints, long wait lists and geographical inequities in service provision (24, 71–73). While specialist multidisciplinary services play a vital role, they cannot meet the needs of all patients. Occupational therapists in community and generalist services report using similar approaches to those used in specialist FND centers (24), but their outcomes have not been adequately evaluated. Future research should therefore examine how occupational therapy interventions can be adapted and delivered in community and non-specialist settings.

Outcome measurement was highly inconsistent. More than 60 different outcome measures were identified across 41 studies, limiting comparison between interventions and settings. Occupational therapy-specific assessment tools were used infrequently. The Canadian Occupational Performance Measure (COPM) was the most frequently reported occupational therapy outcome measure, but it was used in only eight studies. Although the COPM is standardized and widely used in rehabilitation settings, it is not norm referenced and has not yet been validated specifically in people with FND, but it has been identified as a useful tool by expert occupational therapists working in the field (22, 74). The complexities of outcome measurement extend beyond occupational therapy. Additional challenges include the heterogeneous and fluctuating nature of FND symptoms, the often poor alignment between objectively assessed impairment and lived disability, and the lack of validated FND-specific measures capable of capturing change across the range of FND symptoms (75–77). A systematic review of outcome measurement in FND similarly identified substantial variability in outcome selection, with measures most often assessing symptom change, healthcare use, quality of life and general functioning (75). Importantly, this scoping review found that outcomes central to occupational therapy practice such as occupational engagement and participation in vocational and avocational roles, were rarely reported using validated tools. This raises uncertainty as to whether current outcome measurement in FND adequately reflects the aims of occupational therapy or the outcomes most meaningful to patients. Evidence that patients, carers and clinicians may differ in what they view as meaningful outcomes further supports the need to reconsider outcome selection (78). Furthermore, recent randomized controlled trials have shown improvements in secondary outcomes despite non-significant change in the selected primary outcome, raising the possibility that current methods of assessing intervention efficacy may not capture important and clinically meaningful changes (79). Taken together, these findings support the need for consistent and increased use of occupational therapy-specific measures alongside other validated rehabilitation measures to better capture occupational therapy's contribution to FND rehabilitation.

Reported treatment outcomes were generally favorable, with improvements described in symptom severity, physical function, mental health and quality of life. However, controlled evidence was limited to one randomized study, while most findings were derived from uncontrolled observational studies. Occupational therapy was also almost always delivered as part of multidisciplinary rehabilitation, meaning that improvements cannot be attributed specifically to occupational therapy. Study heterogeneity, inconsistent outcome measurement, attrition at follow-up and possible selection bias in who accessed and completed rehabilitation further limit conclusions about effectiveness and longer-term outcomes.

The only occupational therapy-specific FND intervention study identified was a sensory modulation programme, which reported discharge gains in symptom severity, symptom management and occupational performance. Although these early findings are promising, most patients also received concurrent therapies, making the unique effect of occupational therapy difficult to differentiate (27). Nevertheless, these findings suggest that occupational therapy sensory modulation interventions warrant further evaluation in people with FND.

Attempting to isolate the effectiveness of occupational therapy as a profession is neither straightforward nor consistent with how rehabilitation is delivered in practice. A more informative approach to evaluating occupational therapy may be a mechanism first approach, whereby occupational therapy components such as activity focused retraining, sensory modulation and vocational rehabilitation are manualised and evaluated, with fidelity and proximal, mechanism focused occupational therapy outcomes embedded within broader rehabilitation programmes. Rather than asking ‘Which profession is effective?', future research should consider ‘which occupational therapy components are effective, for whom, in what context, and through which mechanisms?'. This shift would strengthen understanding of occupational therapy's contribution within multidisciplinary rehabilitation while supporting the development of more clearly defined and reproducible interventions.

## Limitations

This scoping review has several limitations. While formal critical appraisal of methodological quality was not undertaken, this is consistent with JBI methodology for scoping reviews and reflects the aim of mapping the evidence rather than formally evaluating effectiveness. Study selection was primarily undertaken by one reviewer, with a second reviewer independently screening 10% of titles/abstracts and full texts. Although duplicate independent screening of all records is recommended for JBI scoping reviews, this pragmatic approach was adopted because of resource constraints and is acknowledged as a limitation of the review. Only articles published in English were included, and a small number of potentially relevant papers could not be accessed. However, as these were not randomized or high-level evidence, their exclusion is unlikely to have substantially influenced the overall findings of the review. Generalisability of findings is also unclear, as most studies were conducted in specialist centers and patient populations were highly selected and heterogeneous. Intervention descriptions were often sparse, with limited detail about the specific role of occupational therapy within multidisciplinary programmes. As occupational therapy was almost always delivered as part of team-based rehabilitation, its independent effects could not be isolated. Importantly, this reflects not only a limitation of the review, but also a limitation of the current evidence base, in which profession specific intervention reporting remains insufficient to distinguish the true contribution of occupational therapy from that of the wider multidisciplinary team. Considerable variability in treatment composition and follow-up, coupled with the use of more than 60 different outcome measures further restricted comparison and synthesis across studies. Although some promising evidence exists from large cohort studies and a single randomized trial, there remains a lack of high-quality prospective research to draw firm conclusions about the effectiveness of FND rehabilitation involving occupational therapy. Finally, the review was restricted to published intervention studies and therefore may not capture the full range of occupational therapy interventions delivered in routine clinical practice.

## Conclusions

This review identified recurring components of occupational therapy interventions used in FND rehabilitation research and highlighted important gaps in intervention reporting, outcome measurement and evaluation. There is a growing body of literature describing rehabilitation for FND involving occupational therapy, however, current evidence cannot determine the independent effectiveness of occupational therapy or identify which components contribute more strongly to clinical change. Future research should prioritize the development and evaluation of occupational therapy interventions, including sensory-modulation, vocational rehabilitation and interventions delivered in non-specialist settings. Studies should examine the proposed mechanisms through which occupational therapy interventions may contribute to change, assess treatment fidelity and corresponding proximal outcomes. Once intervention components are better defined, their effects should be tested in robust prospective controlled trials. Incorporating patient perspectives throughout will be essential to ensure that occupational therapy interventions remain relevant and transferable across practice settings.

## Data Availability

The original contributions presented in the study are included in the article/Supplementary material, further inquiries can be directed to the corresponding author.
