# Supplementary material for: Occupational therapy interventions for functional neurological disorder: a scoping review
Source: Front Neurol. 2026 Oct 30;17:1926640. doi: 10.3389/fneur.2026.1926640 (PMC13759413; doi:10.3389/fneur.2026.1926640)
Supplement: Supplementary file 1 [file Supplementary_file_1.docx]

# Supplementary Material

1. Supplementary Material 1: Search Strategy
2. Supplementary Material 2: Summary of Full-text Articles Excluded Following Eligibility Assessment
3. Supplementary Table 1: Detailed study characteristics and intervention outcomes (multidisciplinary rehabilitation)
4. Supplementary Table 2: Detailed study characteristics and intervention outcomes (Occupational Therapy)
5. Supplementary Table 3: Commonly used standardised outcome measures used in rehabilitation for FND

## 1. Search Strategy

**Link:**
[Click to run search](https://eur01.safelinks.protection.outlook.com/?url=https%3A%2F%2Fovidsp.ovid.com%2Fovidweb.cgi%3FT%3DJS%26NEWS%3DN%26PAGE%3Dmain%26SHAREDSEARCHID%3D2Ty8a83KJr6AV5wF5oRXxegEDJcRP7Xw3kASPdOx9jke1dgQ5FCz7zsG8KDu22F1b&data=05%7C02%7Cclare.nicholson.24%40ucl.ac.uk%7C735930f4313048f47c9b08dd96aebfdb%7C1faf88fea9984c5b93c9210a11d9a5c2%7C0%7C0%7C638832399455548599%7CUnknown%7CTWFpbGZsb3d8eyJFbXB0eU1hcGkiOnRydWUsIlYiOiIwLjAuMDAwMCIsIlAiOiJXaW4zMiIsIkFOIjoiTWFpbCIsIldUIjoyfQ%3D%3D%7C0%7C%7C%7C&sdata=Lq%2Fq6%2B0POFAiTJRF9fqEfoRmS3rDF82a5a2IuGwlrJM%3D&reserved=0)
The above Jumpstart will only work for users who have access to this specific database.


**Database:**
Embase <1974 to 2025 May 16>
Ovid MEDLINE(R) ALL <1946 to May 16, 2025>
APA PsycInfo <1806 to May 2025 Week 2>

| **#** | **Query** | **Results from 19 May 2025** |
| --- | --- | --- |
| 1 | (Occupational Therap* or Physical Therap*).mp. [mp=ti, ab, hw, tn, ot, dm, mf, dv, kf, fx, dq, bt, nm, ox, px, rx, an, ui, sy, ux, mx, tc, id, tm] | 212,435 |
| 2 | ((Multidisciplinary or interdisciplinary) adj2 (treatment or program*)).mp. [mp=ti, ab, hw, tn, ot, dm, mf, dv, kf, fx, dq, bt, nm, ox, px, rx, an, ui, sy, ux, mx, tc, id, tm] | 39,383 |
| 3 | 1 or 2 | 249,827 |
| 4 | (Functional neurological adj3 disorder*).mp. [mp=ti, ab, hw, tn, ot, dm, mf, dv, kf, fx, dq, bt, nm, ox, px, rx, an, ui, sy, ux, mx, tc, id, tm] | 3,177 |
| 5 | (Functional neurological adj3 symptom*).mp. [mp=ti, ab, hw, tn, ot, dm, mf, dv, kf, fx, dq, bt, nm, ox, px, rx, an, ui, sy, ux, mx, tc, id, tm] | 897 |
| 6 | (Conversion disorder* or Psychogenic or Medical* unexplained or Hysteria or Dissociative Disorder*).mp. [mp=ti, ab, hw, tn, ot, dm, mf, dv, kf, fx, dq, bt, nm, ox, px, rx, an, ui, sy, ux, mx, tc, id, tm] | 75,077 |
| 7 | (Functional movement disorder* or Functional Motor symptom*).mp. [mp=ti, ab, hw, tn, ot, dm, mf, dv, kf, fx, dq, bt, nm, ox, px, rx, an, ui, sy, ux, mx, tc, id, tm] | 1,545 |
| 8 | 4 or 5 or 6 or 7 | 76,949 |
| 9 | 3 and 8 | 925 |
| 10 | remove duplicates from 9 | 687 |

https://ovidsp.ovid.com/ovidweb.cgi?T=JS&NEWS=N&PAGE=main&SHAREDSEARCHID=2Ty8a83KJr6AV5wF5oRXxegEDJcRP7Xw3kASPdOx9jke1dgQ5FCz7zsG8KDu22F1b

### Updated Search December 2025

**Link:**
[Click to run search](https://eur01.safelinks.protection.outlook.com/?url=https%3A%2F%2Fovidsp.ovid.com%2Fovidweb.cgi%3FT%3DJS%26NEWS%3DN%26PAGE%3Dmain%26SHAREDSEARCHID%3DTPzvIZalIWNRFAtiimvGkxqLRUdn5xPzjVojHGawVqLDkGIoCbOT7PyWq3iwHv1s&data=05%7C02%7Cclare.nicholson.24%40ucl.ac.uk%7C19f4ef27de3c4ec1152a08de33e8db1d%7C1faf88fea9984c5b93c9210a11d9a5c2%7C0%7C0%7C639005272370681412%7CUnknown%7CTWFpbGZsb3d8eyJFbXB0eU1hcGkiOnRydWUsIlYiOiIwLjAuMDAwMCIsIlAiOiJXaW4zMiIsIkFOIjoiTWFpbCIsIldUIjoyfQ%3D%3D%7C0%7C%7C%7C&sdata=LvmL%2B3F8AgDOB%2FqDED1avXw1%2BsfrrjkpWD%2BZJ7K%2BIt4%3D&reserved=0)
The above Jumpstart will only work for users who have access to this specific database.


**Database:**
Embase <1974 to 2025 December 03>
Ovid MEDLINE(R) ALL <1946 to December 04, 2025>
APA PsycInfo <1806 to December 2025 Week 1>

| **#** | **Query** | **Results from 5 Dec 2025** |
| --- | --- | --- |
| 1 | (Occupational Therap* or Physical Therap*).mp. [mp=ti, ab, hw, tn, ot, dm, mf, dv, kf, fx, dq, bt, nm, ox, px, rx, an, ui, sy, ux, mx, tc, id, tm] | 225,619 |
| 2 | limit 1 to yr="2025 -Current" | 12,922 |
| 3 | ((Multidisciplinary or interdisciplinary) adj2 (treatment or program*)).mp. [mp=ti, ab, hw, tn, ot, dm, mf, dv, kf, fx, dq, bt, nm, ox, px, rx, an, ui, sy, ux, mx, tc, id, tm] | 41,534 |
| 4 | limit 3 to yr="2025 -Current" | 2,645 |
| 5 | 2 or 4 | 15,468 |
| 6 | (Functional neurological adj3 disorder*).mp. [mp=ti, ab, hw, tn, ot, dm, mf, dv, kf, fx, dq, bt, nm, ox, px, rx, an, ui, sy, ux, mx, tc, id, tm] | 4,716 |
| 7 | limit 6 to yr="2025 -Current" | 613 |
| 8 | (Functional neurological adj3 symptom*).mp. [mp=ti, ab, hw, tn, ot, dm, mf, dv, kf, fx, dq, bt, nm, ox, px, rx, an, ui, sy, ux, mx, tc, id, tm] | 953 |
| 9 | limit 8 to yr="2025 -Current" | 95 |
| 10 | (Conversion disorder* or Psychogenic or Medical* unexplained or Hysteria or Dissociative Disorder*).mp. [mp=ti, ab, hw, tn, ot, dm, mf, dv, kf, fx, dq, bt, nm, ox, px, rx, an, ui, sy, ux, mx, tc, id, tm] | 76,436 |
| 11 | limit 10 to yr="2025 -Current" | 2,012 |
| 12 | (Functional movement disorder* or Functional Motor symptom*).mp. [mp=ti, ab, hw, tn, ot, dm, mf, dv, kf, fx, dq, bt, nm, ox, px, rx, an, ui, sy, ux, mx, tc, id, tm] | 1,696 |
| 13 | limit 12 to yr="2025 -Current" | 177 |
| 14 | 7 or 9 or 11 or 13 | 2,317 |
| 15 | 5 and 14 | 83 |
| 16 | remove duplicates from 15 | 64 |

https://ovidsp.ovid.com/ovidweb.cgi?T=JS&NEWS=N&PAGE=main&SHAREDSEARCHID=TPzvIZalIWNRFAtiimvGkxqLRUdn5xPzjVojHGawVqLDkGIoCbOT7PyWq3iwHv1s

### Updated Search April 2026

**Link:**
[Click to run search](https://eur01.safelinks.protection.outlook.com/?url=https%3A%2F%2Fovidsp.ovid.com%2Fovidweb.cgi%3FT%3DJS%26NEWS%3DN%26PAGE%3Dmain%26SHAREDSEARCHID%3D18p3HAc45P8L0utBCZx6RcxV8no86B65NSW0AjmtFuYZFECohKrA6017aSRf9HfmD&data=05%7C02%7Cclare.nicholson.24%40ucl.ac.uk%7Ced491fbc57154aecadc308dea071e5db%7C1faf88fea9984c5b93c9210a11d9a5c2%7C0%7C0%7C639124608238831444%7CUnknown%7CTWFpbGZsb3d8eyJFbXB0eU1hcGkiOnRydWUsIlYiOiIwLjAuMDAwMCIsIlAiOiJXaW4zMiIsIkFOIjoiTWFpbCIsIldUIjoyfQ%3D%3D%7C0%7C%7C%7C&sdata=jWILL6RW0cQbISrsxNt%2B68cYUUXOITrFS%2BQLuiF47aY%3D&reserved=0)
The above Jumpstart will only work for users who have access to this specific database.


**Database:**
Embase <1974 to 2026 April 20>
Ovid MEDLINE(R) ALL <1946 to April 21, 2026>
APA PsycInfo <1806 to April 2026 Week 2>

| **#** | **Query** | **Results from 22 Apr 2026** |
| --- | --- | --- |
| 1 | (Occupational Therap* or Physical Therap*).mp. [mp=ti, ab, hw, tn, ot, dm, mf, dv, kf, fx, dq, bt, nm, ox, px, rx, ui, sy, ux, mx, tc, id, tm] | 231,013 |
| 2 | limit 1 to yr="2025 -Current" | 17,547 |
| 3 | ((Multidisciplinary or interdisciplinary) adj2 (treatment or program*)).mp. [mp=ti, ab, hw, tn, ot, dm, mf, dv, kf, fx, dq, bt, nm, ox, px, rx, ui, sy, ux, mx, tc, id, tm] | 42,877 |
| 4 | limit 3 to yr="2025 -Current" | 3,820 |
| 5 | 2 or 4 | 21,226 |
| 6 | (Functional neurological adj3 disorder*).mp. [mp=ti, ab, hw, tn, ot, dm, mf, dv, kf, fx, dq, bt, nm, ox, px, rx, ui, sy, ux, mx, tc, id, tm] | 5,069 |
| 7 | limit 6 to yr="2025 -Current" | 923 |
| 8 | (Functional neurological adj3 symptom*).mp. [mp=ti, ab, hw, tn, ot, dm, mf, dv, kf, fx, dq, bt, nm, ox, px, rx, ui, sy, ux, mx, tc, id, tm] | 1,004 |
| 9 | limit 8 to yr="2025 -Current" | 141 |
| 10 | (Conversion disorder* or Psychogenic or Medical* unexplained or Hysteria or Dissociative Disorder*).mp. [mp=ti, ab, hw, tn, ot, dm, mf, dv, kf, fx, dq, bt, nm, ox, px, rx, ui, sy, ux, mx, tc, id, tm] | 77,436 |
| 11 | limit 10 to yr="2025 -Current" | 2,819 |
| 12 | (Functional movement disorder* or Functional Motor symptom*).mp. [mp=ti, ab, hw, tn, ot, dm, mf, dv, kf, fx, dq, bt, nm, ox, px, rx, ui, sy, ux, mx, tc, id, tm] | 1,806 |
| 13 | limit 12 to yr="2025 -Current" | 265 |
| 14 | 7 or 9 or 11 or 13 | 3,291 |
| 15 | 5 and 14 | 123 |
| 16 | remove duplicates from 15 | 94 |
| 17 | limit 16 to yr="2025 -Current" | 94 |

<https://ovidsp.ovid.com/ovidweb.cgi?T=JS&NEWS=N&PAGE=main&SHAREDSEARCHID=18p3HAc45P8L0utBCZx6RcxV8no86B65NSW0AjmtFuYZFECohKrA6017aSRf9HMotor>

### Updated Search July 2026

The database searches were updated in July 2026 using the original search strategy without date restrictions. The updated search included Ovid MEDLINE(R) ALL (1946 to 27 July 2026), Embase (1974 to 24 July 2026) and APA PsycInfo (1806 to July 2026 Week 3). An equivalent search strategy was also undertaken in CINAHL Plus (EBSCOhost) on 28 July 2026.

**Ovid MEDLINE(R) ALL, Embase and APA PsycInfo search strategy**

1. (Occupational Therap* or Physical Therap*).mp.

2. ((Multidisciplinary or interdisciplinary) adj2 (treatment or program*)).mp.

3. (Functional neurological adj3 disorder*).mp.

4. (Functional neurological adj3 symptom*).mp.

5. (Conversion disorder* or Psychogenic or Medical* unexplained or Hysteria or Dissociative Disorder*).mp.

6. (Functional movement disorder* or Functional motor symptom*).mp.

7. 1 or 2

8. 3 or 4 or 5 or 6

9. 7 and 8

**CINAHL Plus (EBSCOhost) search strategy**

Search date: 28 July 2026

| **#** | **Query** | **Results** |
| --- | --- | --- |
| 1 | (Occupational Therap* OR Physical Therap*) OR ((Multidisciplinary OR interdisciplinary) N2 (treatment OR program*)) |  |
| 2 | (Functional neurologic* N3 disorder*) OR (Functional neurologic* N3 symptom*) OR (Conversion disorder* OR Psychogenic OR Medical* unexplained OR Hysteria OR Dissociative Disorder*) OR (Functional movement disorder* OR Functional motor symptom*) |  |
| 3 | 1 AND 2 | 101 |
| 4 | Records remaining following de-duplication against the updated Ovid MEDLINE(R) ALL, Embase and APA PsycInfo search results | 33 |

**Search syntax**

(XB ("Occupational Therap*" OR "Physical Therap*") OR XB ((Multidisciplinary OR interdisciplinary) N2 (treatment OR program*))) AND (XB ("Functional neurologic*" N3 disorder*) OR XB ("Functional neurologic*" N3 symptom*) OR XB ("Conversion disorder*" OR Psychogenic OR "Medical* unexplained" OR Hysteria OR "Dissociative Disorder*") OR XB ("Functional movement disorder*" OR "Functional motor symptom*"))

## 2. Summary of Full-text Articles Excluded Following Eligibility Assessment

| **Excluded Papers** | |
| --- | --- |
| **Paper** | **Reason for exclusion after full text review** |
| Mueller-Braunschweig H, Moehlen K. Report on the in-patient treatment of a patient with psychogenic seizures with special consideration of nonverbal forms of therapy. Psyche. 1980;34(12):1073–91. | Unable to access |
| De Mol J, Retif J, Aerens C. Post-traumatic conversion hysteria: A case report of hysterical paraplegia. Acta Psychiatr Belg. 1981;81(1):46–56. | Unable to access |
| Klein MJ, Kewman DG, Sayama M. Behavior modification of abnormal gait and chronic pain secondary to somatization disorder. Arch Phys Med Rehabil. 1985;66(2):119–22 | Unable to access |
| Delargy MA, Peatfield RC, Burt AA. Successful rehabilitation in conversion paralysis. BMJ. 1986;292(6537):1730–1. | Ineligible population (no OT) |
| Teasell R, Shapiro A. Strategic Behavioural Intervention In The Treatment of Chronic Nonorganic Motor Disorders. American Journal of Physical Medicine and Rehabilitation. 1994; 73(1): 44-50. | Ineligible population (no OT) |
| Shapiro AP, Teasell RW. Strategic-behavioural intervention in IP rehabilitation of non-organic Motor disorders. NeuroRehabilitation. 1997;8(3):183–92. | Ineligible population (no OT) |
| Watanabe TK, O’Dell MW, Togliatti TJ. Diagnosis and rehabilitation strategies for patients with hysterical hemiparesis: A report of four cases. Arch Phys Med Rehabil. 1998;79(6):709–14. | Ineligible population (no OT) |
| Moene F, Hoogduin K, Van Dyck R. The IP treatment of patients suffering from (Motor) conversion symptoms: A description of eight cases. International Journal of Clinical and Experimental Hypnosis. 1998; 2: 171-190. | Ineligible population (no OT) |
| Schwartz AC, Calhoun AW, Eschbach CL, Seelig BJ. Treatment of conversion disorder in an African American woman: cultural and social considerations. Am J Psychiatry. 2001;158(9):1385–91. | Ineligible population (no OT) |
| Moene F, Spinhoven P, Hoogduin et al. A Randomised Controlled Clinical Trial on the Additional Effect of Hypnosis in a Comprehensive Treatment Programme for In-Patients with Conversion Disorder of the Motor Type. Psychotherapy and Psychosomatics. 2002; 71: 66-76. | Ineligible population (no OT) |
| Ness D. Physical therapy management for conversion disorder: case series. J Neurol Phys Ther. 2007;31(1):30–9. | Ineligible population (no OT) |
| Rayport SF, Schell CA. Diagnostic and therapeutic challenge of nonepileptic seizures: interdisciplinary approach. Epilepsy Behav Case Rep. 2014;2(1):60–3. | Ineligible population (no OT) |
| Stephen CD, Sharma N, Callahan J, et al. Functional dystonia with associated FND: diagnostic and therapeutic challenges. Harv Rev Psychiatry. 2017;25(5). | Ineligible population (no OT) |
| Ranford J, Perez D, MacLean J. Additional occupational therapy considerations for functional neurological disorders: a potential role for sensory processing. CNS Spectrums. 2018; 23: 194-195 | Ineligible study design (no intervention outcomes) |
| Graham CD, O'Hara DJ, Kemp S. A case series of acceptance and commitment therapy (ACT) for reducing symptom interference in functional neurological disorders. Clin Psychol Psychother. 2018;25:489–496. | Ineligible population (no OT) |
| Florentin AS, Fingerman K. Multi-disciplinary treatment of a patient with Functional Neurological Symptoms Disorder hospitalized at a rehabilitation center – A case study. *Journal of the Israeli Physical Therapy Society (JIPTS).* 2018;20(2):35–44. | Not written in English |
| Pourkalbassi D, Patel P, Espinosa PS. Conversion disorder: case report of a patient with non-epileptic seizures. Cureus. 2019;11(1):e3902. | Ineligible population (no OT) |
| Maggio JB, Ospina JP, Callahan J, et al. Outpatient physical therapy for FND: feasibility and naturalistic outcomes study. J Neuropsychiatry Clin Neurosci. 2020;32(1):85–9. | Ineligible population (no OT) |
| Hsieh Y, Deshpande SN. Therapy-led multidisciplinary programme for treatment-resistant functional fixed dystonia. BMJ Case Rep. 2020;13(7). | Ineligible population (no OT) |
| Ranford J, MacLean J, Alluri P et al. Sensory Processing Difficulties in Functional Neurological Disorder: A Possible Predisposing Vulnerability? Psychosomatics. 2020; 61 (4): 343-352. | Ineligible study design (no intervention outcomes) |
| LeVan G, McDade M. Using an Occupation-Based and Interdisciplinary Approach to Functional Neurological Disorder: A Case Study. The American Journal of Occupational Therapy. 2022; 76, Supplement 1. | Ineligible study design (conference abstract only) |
| Aslantaş HO, Sonel Tur B, Şenol Ç, Togay Işıkay C, Duman B, Devrimci Özgüven H, Kalın AS**.** A case with a history of cerebrovascular disease: recurrent stroke or a pseudoneurological disorder? *Journal of Physical Medicine and Rehabilitation Sciences.* **2022**;**25**(1):127–132. | Not written in English |
| MacLean J, Finkelstein S, Paredes-Echeverii et al. 2022. Sensory Processing Difficulties in Patients with Functional Neurological Disorder: Occupational Therapy Management Strategies and Two Cases. Seminars in Pediatric Neurology. 2022; 41: 1-9. | Ineligible study design (no intervention outcomes) |
| Pepper E, Mohan A, Butcher K et al. Functional neurological disorders: an Australian interdisciplinary perspective. Journal. 2022; 216(10): 501-503 | Ineligible study design (no intervention outcomes) |
| Polich G, Zalanowski S, Maney J et al. Development of an IP rehabilitation pathway for Motor functional neurological disorders: Initial reflections. NeuroRehabilitation. 2022; 50: 231.243 | Ineligible study design (no intervention outcomes) |
| Polich G, Thompson J, Molton I et al. Intensive rehabilitation for functional Motor disorders (MOTOR) in the United States: A review. NeuroRehabilitation. 2022; 50: 245-254. | Ineligible study design (no intervention outcomes) |
| Watson M, Borland H, Baker S, et al. Influence of telehealth on engagement in a multidisciplinary NES clinic during COVID-19. Epilepsy Behav. 2022;13. | Ineligible population (no OT) |
| Gilio L, Galifi G, Centonze D, Stampanoni Bassi M. Overlap between long COVID and FND: Case report. Front Neurol. 2022;12. | Ineligible population (no OT) |
| Nguyen AT, Hemphill S, Donahue B, et al. Use of virtual reality for targeted physical rehabilitation: Case report on managing functional Motor disorder. J Pediatr Rehabil Med. 2023;16(2):415–23. | Ineligible population (no OT, subjects under age of 18) |
| Palmer DDG, Gamble M, Higgins M, Maley J, Watson E. Outcomes of an integrated multidisciplinary clinic for FND. Mov Disord Clin Pract. 2023;10(6):967–73. | Ineligible population (no OT) |
| Bailey C, Agrawal N, Cope S, et al. Illness perceptions, stigma and engagement in FND: role of multidisciplinary group education sessions. BMJ Neurol Open. 2024;6(1). | Ineligible population (no OT) |
| Mowry H, Perez E, Stillwell A, Shearin S. Physical therapy evaluation and treatment of functional movement disorders: A case series. Physiotherapy Theory and Practice. 2025; 41 (11): 2468-2479. | Ineligible population (no OT) |
| Houtveen J, van Eck van der Sluijs, Jan Herremans P, Geenen R. Treatment-related changes during and after IP treatment for refractory somatic symptom disorder or functional neurological symptom disorder: Testing the applicability of a new approach for analyzing routine outcome monitoring data. Journal of Psychiatric Research. 2025; 185: 138-145. | Ineligible population (no OT) |
| Sandri A, Di Vico IA, Geroin C, Bombieri F, Vandelli V, Tinazzi M, Gandolfi M. Does duration Matter? Evaluating the impact of short- and long-term telemedicine in functional Motor disorders. Parkinsonism and Related Disorders. 2025; 137:1-5. | Ineligible population (no OT) |
| Moscovich M, Estupinan D, Qureshi M, Okun MS. Psychogenic gait and movement disorders: film review. Tremor Other Hyperkinet Mov. 2013. | Ineligible study design |
| O’Neal MA, Dworetzky BA. Caring for patients with FND: case-oriented review. Neurologist. 2016;21(3):39–43. | Ineligible study design (no intervention outcomes) |
| McKee K, Glass S, Adams C, Stephen CD, et al. IP assessment and management of Motor FND: interdisciplinary perspective. Psychosomatics. 2018;59(4):358–68. | Ineligible study design (no intervention outcomes) |
| O’Neal MA, Baslet G. Treatment for patients with FND: integrated approach. Am J Psychiatry. 2018;175(4):307–14. | Ineligible study design (no intervention outcomes) |
| Pasquale BGD, Teive HAG, Pasquale LFA, von der Heyde MD. Management of functional seizures and movement disorder: cross-sectional study. Neuropsychiatr Dis Treat. 2022;18:2121–31. | Ineligible study design |
| Lu SY, Yang YW, Liu Y, Wang G, Xie Q, Wang JX. Advances on rehabilitation treatment of functional Motor disorder. Chin J Contemp Neurol Neurosurg. 2023;23(12):1097–1102. | Ineligible study design (no intervention outcomes) |
| Yin D, Wang G, Wang JX. Diagnosis and treatment of functional limb weakness. Chin J Contemp Neurol Neurosurg. 2023;23(10):924–8. | Ineligible study design (no intervention outcomes) |
| Stone J, Carson A. Multidisciplinary Treatment for Functional Movement Disorder. Continuum (Minneap Minn). Movement Disorders. 2025; 31(4): 1182-1196. | Ineligible study design (no intervention outcomes) |
| Trierweiler E, Hubsch C, Berney A. Functional neurological disorders: Integrated, patient-centered neuropsychiatric approach. Revue Médicale Suisse. 2026; 22(949): 305–310. | Not written in English |

## 3. Table 1: Detailed study characteristics and intervention outcomes (multidisciplinary rehabilitation)

| ***Ref*** | ***Design*** | ***Participant Characteristics*** | ***n*** | ***Setting*** | ***Intervention Description*** | ***Delivery mode, intensity and duration*** | ***Data collection methods*** | ***Patient clinical Outcomes*** | ***Key OT Findings*** |
| --- | --- | --- | --- | --- | --- | --- | --- | --- | --- |
| Foong et al., 2026 (63) | Retrospective cohort study | *Sex:* 71 (71%) female, 29 (29%) male.  *Age:* 42.3+11.8 (mean / SD)  *FND Type:* Mixed FNS + 51 (51%) presenting with FND + predominant pain  *Symptom Duration:* Not reported | 100 | IP | *Clincian Type:* neuropsychiatry, neurology, mental health nursing, psychology, PT, OT, SALT  *Intervention:*  Aims to reduce symptoms, improve functional performance and health status. Weekly goal setting and regular input from therapists. Detailed programme content not fully described previously outlined in Demartini et al., 2014 and Saifee et al., 2012 (same setting). | MDT  *Intervention period:* 4 weeks  *Intensity*: not specified | COPM  EQ-5D-5L  PHQ-9,  GAD-7  *Follow-up period:* Not reported | Change scores represent admission to discharge:  **COPM (n=83):** Performance +2.8–3.5; Satisfaction +4.2–4.5 (clinically meaningful in both groups; NS between groups) **PHQ-9 (n=47):** −8.19 vs −3.5 (p = 0.026) **GAD-7 (n=47):** −7.11 vs −1.93 (p = 0.008) **EQ5D5L (n=90):** +20–25 (NS between groups) **Pain/discomfort:** −1 both groups (NS) | OT role not described  OT intensity not described  Standardised OT measures described |
| Rush et al., 2025 (46) | Prospective cohort study | *Sex:*22 (48.9%) male, 23 (51.1%) female  *Age:* 48.2 +14.9 years (mean / SD)  *FND Type:* Motor symptoms  *Symptom Duration:* Mean: 42.6 months, Median; 16 months. | 45 | OP | *Clincian Type:* PMR physician, PT, OT  *Intervention:*  5 consecutive days of intensive out-patient physiotherapy, OT focused on deconstructing abnormal movement /postures, normal movement re-training.  Relaxation exercises, introduction of cognitive and environmental challenges to promote carry over into real world scenarios. Physican follow-up post programme completion. | MDT  *Intervention period:*  5 days  *Intensity:*  OT/PT – 3-4 one-hour sessions daily over 5 days | COPM  PSFS  GROC  IUS-12  *Follow-up period:*  Reported but timing not specified | Change scores represent admission to discharge:  **COPM:** Performance +3.86, Satisfaction +5.20 (clinically meaningful) **PSFS:** +10.43 **GROC:** +3.49 (moderate improvement) **IUS-12:** −5.27 (reduced intolerance of uncertainty) | OT role partially described.  OT intensity described  Standardised OT measures described. |
| Gilmour et al., 2025 (45) | Case series | *Sex:* Not reported  *Age:* Not reported  *FND Type:* Motor  *Symptom Duration:* Not reported | 20 | OP | *Clinician Type:* neurology, neuropsychiatry, nursing, clinical psychology, neuropsychology, PT, OT, social work, SALT.  *Intervention:*  Initial integrated assessment (Neurology, neuropsychiatry, nursing).  5-day intensive MDT (including OT) outpatient rehabilitation programme tailored to patient-specific goals.  Virtual psychoeducation groups (neuropsychology).  Follow-up visits: 6-week telephone follow-up and drop-in visits (PT, neurology, nursing)  Discharge review at 1 year (Neurology, nursing). | MDT  *Intervention period:*  5 days embedded within a 1-year pathway.  *Intensity:*  PT: 1hr daily (5hrs)  SLT: 3hrs over 5 days  OT: 2hrs over 5 days  SW: 1hr over 5 days | COPM  EQ-5D-5L  Goal achievement  Involvement in decisions about care  *Follow-Up period:* 6 weeks and 12 months | COPM (n=20): mean performance: +2.9 [SD 2.1], satisfaction: +3.9 [SD 2.4]  EQ5D5L (n=20): mean + 10.2 [SD 17.6]  Goal achievement (n=15): 93% mostly or entirely agreed.  Involvement in decisions about care (n=15): 100% mostly or entirely agreed. | OT role not specifically described.  OT intensity described.  Standardised OT measures described. |
| Le Perf et al., 2025 (66) | Retrospective cohort study | *Sex:* 14 (70%) female, 6 (30%) male  *Age:*29-76 (median 65)  *FND Type:* PPPD  *Symptom Duration:* Not reported | 20 | IP | *Clinician Type:* PT, OT, psychologists, physical activity instructor, psychoMotor therapists, otolaryngologists  *Intervention:* Vestibular rehabilitation, balance and functional mobility training, education, adapted physical activity, psychoMotor therapy. Focus on postural control, symptom management, functional recovery. | MDT  *Intervention period:*  4 weeks  *Intensity:* 5 days per week, daily individual and group sessions | MiniBestest  Sensory Organisation Test (posturography)  Postural Instability Index (PII)  6MWT  Dizziness Handicap Inventory (DHI)  *Follow-up period:* Not reported | Balance (MiniBESTest +5.46, p<.001)  Disability (DHI −18.15, p<.001)  Mobility (6MWT +129.31 m, p<.001)  Postural control (posturography and PII improved) | OT role described  OT intensity described  Standardised OT measures not described |
| Zheng et al., 2025 (51) | Case report | *Sex:* Male  *Age:* 44 years  *FND Type:*  Functional seizures  Functional Tremor  Functional weakness  Speech disturbance  Cognitive dysfunction  *Symptom Duration:* 6 months | 1 | IP | *Clinician Type:*physiatrist, PT, OT, SALT, social work, nursing, psychologist.  *Intervention:*Daily PT, OT, SLT incorporating graded activity practise, psychobehavioural approaches and CBT informed strategies, regular team review and medical management of pain / spasticity. | MDT  *Intervention period:*  8 days  *Intensity:*  3hrs total daily of PT, OT, SLT | Clinician rated level of assistance at admission / discharge e.g. minimal, moderate assist, setup, independent.  Cognitive assessment: MOCA  Speech and language, voice and articulation, fluency assessment – Non-standardised.  *Follow-up period:* Not reported. | Improved independence in ADLs (to independent/supervision level), increased upper and lower limb strength, improved static and dynamic balance for fall prevention, and improved cognitive function including short-term memory and problem-solving. Admission MOCA: 22/30, discharge score not specified. | OT role not specifically described.  OT intensity described.  Standardised OT measures not described. |
| Schneider et al., 2024  (50) | Single-centre retrospective study: retrospectively extracted data from patient files. | *Sex:* 21 (70%) female, 9 (30%) male  *Age:* 43 +14.7 years (mean / SD)  *FND Type:*  Motor symptoms: 13.3%  Dysarthria (6.7%)  Functional seizures 3.3%  Functional hemiparesis (53.3%)  Sensory impairment (60%).  *Symptom Duration:* 27/30 admitted for a first acute episode of FND and 3 patients admitted for FND relapse. | 30 | IP | *Clinician Type:* psychiatry, PT, OT, psychotherapy.  *Intervention:*  Psychoeducation, support therapy, identification of triggers and maintaining factors.  Specific therapy roles not defined. | MDT  *Intervention period:* mean 4 weeks.  *Intensity:*  Psychotherapy once weekly.  Assessment by Psychiatry.  Two daily sessions of PT and one daily session of OT for 30-45 minutes, 5 days per week. | FIM  Work status at admission and follow-up.  OT assessment tools not specified.  *Follow-up period:*  Mean: 36 months. | Outcome was reported as good in 22 participants at discharge with a degree of functional independence for all Motor FIM items and in 21 at FU.  56.7% working at discharge and 40% working at follow-up.  Disability insurance payouts: 43.3 % prior to admission and 60% at FU.  10% experienced significant relapse requiring a second hospitalisation; one after 25 months, one after 27 months and another at 38 months.  Absence of a comorbid psychiatric disorder was a predictive factor for a good outcome at follow-up. | OT role not described.  OT intensity described.  Standardised OT measures not described. |
| Saunders et al., 2024  (40) | Retrospective cohort study | *Sex:* 34 (65%) female, 17 (32%) male, 1 (2%) non-binary  *Age:* Mean 40, range: 21-82 years.  *FND Type:* mixed; 60%, functional seizures; 17%, Motor symptoms; 14%, Cognitive symptoms; 6%, extreme fatigue;4%.  *Symptom Duration:* Mean 9.7 years (SD 8.83; range 1–40) | 52 | IP | Clinician Type: clinical psychology, PT, OT, medic.  *Intervention:*  Formulation, psychoeducation, building therapeutic relationships, goal setting. Individual sessions with clinical psychology, PT and OT. Regular medical review, goal setting sessions, activity groups and social work input if needed. Graded discharge.  OT: activity planning and management, relaxation strategies, building confidence and life skills.  Psychology: CBT approach + dynamic, interpersonal aspects as required.  PT: movement re-training, attentional strategies, education, exercise | MDT  *Intervention period:* Mean 19.4 weeks, range; 3-76 weeks.  *Intensity:* not specified | CGI-I, EQ-5D-5L, EQVAS, FMS, BDI-II, STAI, CDS, IPG, IPQ-R, BBS, 10MTW, TUAG.  OT assessment tools not specified.  *Follow-up period*: Not specified. | 43/52 patients’ global condition had improved on the CGI-I; 12, very much improved, 12, much improved, 19, some improvement, 8, no benefit.  1 patient deteriorated (pre-existing mental health condition).  Measures of mobility, depression and quality of life significantly improved.  Symptoms were experienced as more understandable and less distressing.  Confidence in treatment was predictive of outcome. | OT and MDT roles described.  Therapy intensity not specified.  Standardised OT measures not described. |
| Polich et al., 2024  (48) | Retrospective Cohort study: chart review. | *Sex:* 90.1 % females (2 men)  *Age:* Mean 44 (SD 17.7)  *FND Type:*  Limb weakness 86.4%  Generalised FND 27.3%  Functional paraplegia 18.2%  Functional hemiplegia 36.4%  Gait disorder 95.5%  Speech / swallow/ voice 13.6%  Cognitive symptoms 40.9%  Functional seizures 22.7%  *Symptom Duration:* less than 1 week for nearly 2/3 of the cohort | 22 | IP | *Clinician Type:* physical medicine and rehabilitation, psychology, PT, OT, nursing, social work and case management. Psychiatry and SLT services if required. Neurologic music therapy, aquatic therapy and therapeutic recreation.  *Intervention:*  Music therapy co-delivered by neurologic music therapist and PT using rhythmic auditory stimulation method.  Aquatic therapy co-delivered by PT and OT; exercises for ambulation, stair training, jumping, balance, co-ordination.  Therapeutic recreation sessions offered individually and with PT / OT; included games, sports, arts and crafts, community outings.  PT, OT, SLT consensus recommendations for FND were applied and the MDT were educated about the key principles in an FND education session.  Psychological interventions; skills-based CBT, mindfulness-based psychotherapy for FND.  Weekly individualised FND education session; exploration of triggers, maintaining factors, warning signs, unhelpful thoughts and beliefs.  Self-management plan (FND toolkit) completed prior to discharge.  Patients were referred for community therapy on discharge if required. | MDT  *Intervention period:* 2-weeks  *Intensity:*  Psychotherapy – twice weekly sessions between 30 and 60 mins.  *Intensity:*  3 or more hours of therapy delivered daily over 5 to 6 days per week. Majority of time spent in OT and PT.  45.5% of patients participated in SLT.  Neurologic music therapy – 1 to 4 sessions weekly  Therapeutic recreation – 1-7 sessions weekly | Physical and OT measures on the IRF-PAI.  Independence level for self-care tasks (eating, oral hygiene, toileting hygiene, showering / bathing, dressing, functional transfers.  6-point scale: 1 = dependent, 2 = max assistance, 3 moderate assistance, 4 = supervision or touching assistance, 5 = set-up, 6 = independence.  *Follow-up period:* not specified. | Target IP admission LOS of 2 weeks: approximately met: median: 14.3 % SD 4.6. Range 7 – 25 days.  Statistically significant changes in admission and discharge measures of self-care, transfers, ambulation and balance.  90% of patients showed improvements on the OT summary variable.  95% showed improvements on the PT transfer summary variable.  At discharge, significantly more patients were ambulatory, walking independently and without assistive devices. Berg balance scores improved to a statistically significant degree.  1 /22 patients showed no improvement.  More than 95% of patents were able to be discharged home. | OT role described.  Therapy intensity described.    OT assessment tools specified. |
| McCombs et al., 2024  (27) | Retrospective cohort study | *Sex: 58 women, 19 men*  *Age: mean* 40.3, SD; 14.0.  *FND Type:* Motor symptoms: 57  Functional seizures: 29  Speech: 21.  *Symptom Duration:* 5.7 years, SD 8.6 years | 77 | OP | *Clinician Type:* OT / MDT  11 patients stand-alone OT  45 patients’ concurrent psychotherapy  39 concurrent PT  17 concurrent SLT  *Intervention:*  Treatment based on 8 OT sensory-based treatment modules previously described (10) | MDT: 66  Independent OT: 11  *Intervention period:* 1-6 months.  *Intensity:*  1-hour OT treatment sessions over 1-6 months, starting with once weekly with a reduction in frequency to once every few weeks.  Sessions delivered in person and remotely. | AASP  Primary outcome; clinician-rated improvement rating; somewhat improved, much improved based on information extracted from the discharge or last available treatment note.  *Follow-up period:* not specified. | Patients with FND reported sensory processing patterns with elevated scores; low registration, sensory sensitivity, sensory avoidance compared with normative values.  62% of the patients were “improved” post-sensory-based OT intervention.  Number of treatment sessions positively correlated with clinical improvement. | OT role described.  OT intensity described.  OT assessment tools specified. |
| Callister et al 2023  (43) | Retrospective cohort study | *Sex:* 148 female, 53 male  *Age:* Mean 46  *FND Type:* Motor symptoms  *Symptom Duration:* median 24 months | 201 | OP | *Clinician Type:* neurology, physical medicine and rehabilitation, psychiatry, psychology, OT, PT.  *Intervention:*  Therapeutic screening offered to some patients: screening for eligibility, education about the program, basic education and demonstration of treatment strategies.  Re-training normal automatic movements in line with consensus recommendations for PT and OT.  Education in relaxation exercises, graded movement re-training, and self-management strategies.  Carry-over of taught strategies into functional tasks and different contexts.  Physician follow-up post-discharge. | MDT  *Intervention period:* 5 days  *Intensity:* 3-4 1-hour sessions daily between PT and OT | COPM  GROC  Retrospective investigator-rated scale (1-3 – worse /not improved to significantly improved/resolved).  *Follow-up period:* discharge or within 30 days post-completion. | Substantial improvement in COPM performance and satisfaction scores (mean 3.8 SD 1.9).  GROC (mean 5.5, SD 1.7)  Investigator-rated outcomes – 84% moderately to significantly improved/resolved.  Predictors of improved outcome: participation in pre-admission therapeutic screening, higher number of non-Motor symptoms, shorter MOTOR duration, earlier program entry, lower baseline COPM scores, higher GROC between therapeutic screening and program start.  3% of patients that underwent the screening improved and therefore did not require admission to the program. | OT role described.  OT intensity described.  OT assessment tools specified. |
| Ashrafzdeh 2023  (65) | Case report | *Sex:* Male  *Age:* 28  *FND Type:* Motor symptoms, functional seizures with Cushing’s Disease.  *Symptom Duration:* 6 months | 1 | IP | *Clinician Type:* acute medical team, psychiatry,  endocrinology, neurosurgery, psychology, PT, OT  SLT.  *Intervention:*  Education re: FND  Interdisciplinary approach to rehabilitation.  PT for leg strength, standing, walking.  OT for hand dexterity: writing, drawing, stress ball. | MDT  *Intervention period:* not specified  *Intensity:* not specified | Standardised assessment measures not specified.  *Follow-up period:* Not specified. | Regained hand function (able to eat and write).  After being bedbound for 8 months, patient was able to stand and take steps with assistance.  Reduced seizure frequency.  Improvements in speech quality.  Depressed mood, anhedonia and suicidal ideation decreased, functional symptoms improved. | OT role described.  OT intensity not described.  OT assessment measures not specified. |
| Geary 2022  (49) | Case series | *Sex:* 3 women  *Age:* 28, 21, 41  *FND Type:* Motor symptoms, functional weakness, dizziness, functional seizures.  *Symptom Duration:* 3 days, 4 weeks, not specified. | 3 | IP | *Clinician Type:* PT, OT, physical medicine and rehabilitation doctor, music therapy, psychology and social work.  *Intervention:* Education re: FND.  PT – individualised plan and treatment based on PT consensus recommendations  OT role not described. | MDT  *Intervention period:* 13 to 18 days  *Intensity:*  PT at least 3 times per week  Each received three hours of therapy per week between PT, OT | PT outcome measures only reported: BBS, 10MWT, FGA  OT assessment tools not specified.  *Follow-up period:*  Case study 1 and 3 not specified. Case study 2: ‘a few months after discharge’ | Each patient made functional gains by discharge. Improvements measured by changes in independence with transfers, gait, walking, ability to manage stairs, level of supervision / assistance required. | OT role not described.  OT assessment tools not specified.  Functional abilities outside of mobility were not discussed. |
| Hebb et al 2022  (60) | Case report | *Sex:* Woman  *Age:* 24  *FND Type:* Motor symptoms  *Symptom Duration:* Not specified | 1 | IP | *Clinician Type:* neurology, music therapy, PT, OT, SLT, psychotherapy.  *Intervention:*  Rhythmic auditory stimulation (RAS) delivered by a neurologic music therapist in conjunction with PT.  Cognitive dual tasking distractions  Compensatory strategies used in SLT sessions e.g., schedules, notes.  RAS used to assist with gait re-training alongside PT  OT role not described. | MDT  *Intervention period:* 8 days  *Intensity*: not specified | BBS, DGI, 10MWT  OT specific assessment tools not specified.  *Follow-up period:* 4 years. | Minimal carry over in and between PT sessions until RAS was introduced = overall improvements in gait with resolution of compensatory movements following cures to internalise walking to a beat.  4-year follow-up – gait disorder remained resolved but fatigue and pain limited walking endurance. | OT role not described  OT intensity not described.  OT assessment tools not specified. |
| Reid et al., 2022.  (44) | Retrospective case series | *Sex:* 17 women, 1 male  *Age:* 21 to 71 years, mean: 46.67 years  *FND Type:* Motor symptoms  *Symptom Duration:* Not specified | 18 | OP | *Clinician Type:* PT, OT, SLT  *Intervention:* Autonomic regulation and Motor re-training. Diaphragmatic breathing, vagal nerve stimulation, mindfulness re-training. Motor re-training within functional tasks, whilst incorporating mental imagery. | MDT  *Intervention period:* 5 days.  *Intensity:*  Daily:  90 minutes of PT  90 minutes of OT  30 mins of meditation / mindfulness  60 mins SLT | 6-minute walk test, BBS, TUAG, STS, PSFS, 30-second Arm Curl Test, COPM, BBT.  *Follow-up period*: not specified. | Significantly improved Motor and gait outcomes. Improvements in all outcome measures except for Timed up and Go Test  Improved patient perception of functional performance | OT role described  OT intensity described.  OT assessment tools specified. |
| MacLean et al., 2022  (25) | Case reports | *Sex:* Female, male  *Age:* 23, 42  *FND Type:* Mixed FNS  *Symptom Duration:* 3 years (36 months), 1 year (12 months) | 2 | OP | *Clinician Type:* OT, PT, CBT, SLT.  Case study 1; received PT alongside OT.  Case study 2: received CBT, SLT alongside OT.  *Intervention:*  OT intervention: physical examination, observation of Motor skills, sensory processing assessments (AASP), review of daily routine and impact of symptoms on function.  Individualised sensory-based treatment plan: sensory processing profile, sensory Motor preference checklist, self-regulation strategies, pacing strategies. | MDT  *Intervention period & Intensity:*  Case study 1: OT once every 2 weeks for 2 months (7 sessions). Follow-up at 4 & 8 months.  Case study 2: OT once weekly for 8 weeks (7 sessions). Follow-up period not specified. | AASP  COPM  *Follow-up period:* 4 & 8 months | Case study 1: Increased self-awareness of sensory preferences and tendencies, independent use of sensory strategies.  Improvements in COPM scores: performance by 3.6, satisfaction by 5.  Patient reported improved Motor control, increase stress management.  FNS ‘much improved’ at follow-up.  Case study 2:  Reduced seizure frequency, reduced stress and anxiety, increased community access, improved cognition.  COPM: performance increased by 3.4, satisfaction by 2.2. | OT role described  OT intensity described.  OT assessment tools specified. |
| Schmidt et al 2021  (59) | Prospective cohort study | *Sex:* 14 men, 17 women  *Age:* Median 47 years, range; 19-64  *FND Type:* Motor symptoms  *Symptom Duration:* Median 36 months, range; 3-130 months. | 31 | IP | *Clinician Type:* neurology, CBT, PT, SLT, music therapy, OT, nursing. Lead physician was a neurologist, psychiatrist, and psychotherapist.  *Intervention:*  Physical Therapies – enhancement of body awareness, Motor re-training.  OT role not specifically described. | MDT  *Intervention period:* Range: 10-35 days, median; 21 days  *Intensity:*  10-15 individual and group sessions per week, including 2-3 psychotherapy sessions and 3-4 PT sessions weekly  Sessions: 20-60 mins.  OT intensity not specified | Blinded video rating using the PMDRS, S-FMDRS and CGI-S, patient self-rating.  OT specific assessment tools not described.  *Follow-up period:* average 5 months, range 1-20 months. | Significantly reduced symptoms in patients with MOTOR at discharge and follow-up.  Age correlated with treatment response – older patients showing better improvements.  Disease duration did not correlate with outcome.  Patients who suffered from physical trauma, sexual or physical abuse, had smaller improvements. | OT role not described  OT intensity not described.  OT assessment tools not specified. |
| Hebert et al., 2021.  (58) | Prospective case series with long-term follow-up | *Sex: 14 women, 3 men*  *Age: Mean* 41.76 years  *FND Type:* Motor symptoms  *Symptom Duration:* 3 days to 22 years (144 months) | 17 completed rehabilitation  13 completed a one-year follow-up | IP | *Clinician Type:* physiatry, neurology, psychiatry, psychology, PT, OT and SLT.  *Intervention:* Physical therapies: Motor re-training, identification of symptom triggers, and maintaining factors. Positive movements are re-enforced, abnormal movements ignored.  OT role not specifically described. | MDT  *Intervention period:* Median 7.5 days (range 3-14 days)  *Intensity* of physical therapies not described | CGI, DSSS, GAD-7, PHQ-9, PCL-5.  PT and OT assessment tools: MAS, FGA, BBS, TUG, FIST, FIM Motor scale.  *Follow-up period*: 12 months | Improvements made in function, depression, anxiety, and somatic symptoms at discharge.  At one-year follow-up there had been minimal sustained improvement and worsening psychopathology, reflecting chronic disability despite initial rehabilitative success. | OT role not described.  OT intensity not described. |
| Henry et al 2021  (39) | Case report | *Sex: Female*  *Age: 25*  *FND Type:* Functional seizures and functional weakness  *Symptom Duration:* 84 months | 1 | IP | *Clinician Type:* psychiatry, DBT, pharmacotherapy, OT, PT.  *Intervention:* structured program of weekly activities which the patients help to develop and sometimes lead.  PT – worked on balance and strength + trauma-focused PT  OT role not described. | MDT  *Intervention period:* 12-months  Two cycles of DBT skills training programme + 12 months of one-to-one sessions  For six months post discharge from IP unit: received six months of one-to-one follow-up with DBT  *Intensity* of physical therapies not described | CORE, DERS, GAD-7, PAI-BOR, RFL,  KIMS, PHQ-9, QPR, SWEMWBS.  OT assessment tools not specified.  Follow-up period: Weekly follow-ups with care co-ordinator for 6 months. | Improvements on all outcome measures at discharge and 6-month follow-up.  On discharge, patient was self-harm free, walking unaided and no longer met EUPD criteria.  On admission she used a wheelchair, on discharge she mobilised mostly with a walker, at times unaided indoors, wheelchair for longer distances. Seizure free at discharge. | OT role not described.  OT intensity not described.  OT assessment tools not specified. |
| Joos and Halmer 2020  (57) | Case report | *Sex: Male*  *Age: 64*  *FND Type:* Motor symptoms  *Symptom Duration:* 2 years (24 months) | 1 | IP | *Clinician Type:* PT, OT, individual and group psychotherapies, art therapy.  *Intervention:* Integrated IP rehabilitation, including mirror therapy.  OT used manual therapy to relax muscles alongside mirror therapy | MDT  *Intervention period:* 8 weeks  Independent mirror therapy training – 15-20 mins daily  *Intensity:*  Individual psychotherapy (50 min per week)  Group psychotherapy) three times 50 mins per week)  OT intensity not specified | Not specified  OT assessment tools not specified.  Follow-up period: 2 months. | Tremor substantially decreased after 8 weeks  Improved FMC, improved hand-eye co-ordination.  Improvement sustained at 2-month follow-up. | OT role description lacks detail to guide practice.  OT intensity not specified.  OT assessment tools not specified. |
| Faul et al 2020  (34) | Case Series | *Sex:* 12 women, 2 men mean  *Age: Mean 48.43*  *FND Type:* Motor symptoms  *Symptom Duration:* mean 7.27 years (87.24 months) | 14 | IP | *Clinician Type:* PT, OT, SLT, CBT. Overseen by a physiatrist.  *Intervention:*  Emotional go / No Go fMRI task before and after MoRE treatment: Motor re-training using therapy principles validated for MOTOR. | MDT  *Intervention period:* 5 days  *Intensity:* 3 hours daily of PT, OT, SLT (if necessary) and one hour of CBT with a psychologist | During scanning, patients completed an emotional Go / No Go Task with blocks of fearful, happy and neutral faces. Standardized pre and post-treatment videos were rated for Motor severity using the PMDRS. PMDRS scores before and after treatment were used for whole brain regression.  Patient rated symptom improvement using the CGI.  BDI, STAI, were also completed.  OT assessment tools not specified.  *Follow-up period:* Not reported. | PMDRS scores were significantly reduced after treatment. Patients reported significant improvement in symptoms.  Worse severity prior to treatment was associated with greater primary Motor cortex (M1) activation at baseline and a larger response to treatment.  Globally increased connectivity between bilateral amygdala and preMotor regions was observed following treatment.  Findings suggest that short-term MDT treatment for MOTOR patients improves Motor severity and may have reorganised activity and connectivity in emotional and Motor pathways. | OT role not described.  OT specific assessment tools not used. |
| Petrochilos et al., 2020  (47) | Retrospective case series | *Sex:* 60 females, 18 males  *Age:* 19 -76 (mean: 42.6 years)  *FND Type:* 50% predominant Motor symptoms, 41% functional seizures, 9% sensory or cognitive symptoms, 81% mixed symptoms.  *Symptom Duration:* Mean: 6.5 years (78 months) | 78 | OP | *Clinician Type:* neuropsychiatry, OT, PT, CBT.  *Intervention:*  Group FND education.  Psychiatry: 1:1 sessions: reviewing dx, managing co-morbidities, medications, exploring barriers to progress and onward referrals.  CBT: Personalised explanation of the CBT model based on individualised 3 Ps, build insight and awareness into emotions and triggers, behavioural interventions, make links between behaviours perpetuating maladaptive symptoms or responses to situations / states.  OT: Re-engagement with daily activities. Aim to normalise participation and reduce reliance on equipment and input from others. Self-management principles and the use of graded goal setting. Identification of barriers to function and integration of taught symptom management strategies into daily activities. Fatigue, pain and anxiety management, improving structure and routine, graded activity practise, exploring how cognitive challenges could be reduced, community access, assisting with return to vocational roles; work, education, childcare, volunteering and leisure.  PT: movement re-training. Use of distraction and relaxation strategies. CBT model used to challenge beliefs about the assumed consequences of movement and use of compensatory strategies. Pain management, initiating a graded exercise approach.  Joint working where necessary. All disciplines involved with a relapse prevention planning and setting graded goals for discharge.  Discharge family meeting  6-month review | MDT  *Intervention period:* 5 weeks  *Intensity:*  2 sessions of OT, PT, CBT per week for 5 weeks + 3 individual sessions with psychiatry + MDT review session.  Sessions: up to 1 hour | Clinician rated: HONOS  Patient rated: benefit of program, PHQ15, PHQ9, GAD7, RSE, SPIN, EQ-5D-5L, WSAS, CGI, COPM. Collected at start, discharge, 6-month review.  *Follow-up period:* 6 months. | High levels of disability and psychopathology on admission.  Statistically significant improvements in somatic symptoms as measured on the PHQ15, depression (PHQ9), anxiety (GAD7), health and social functioning (HONOS), function (COPM), health status (EQ-5D-5L) and patient rated clinical global improvement (CGI) (discharge and 6-month review). | OT role described.  OT intensity described.  OT assessment tools described. |
| Jimenez et al 2019  (64) | Retrospective case series | *Sex:* 33 women, 17 men  *Age:* mean 42.53 years  *FND Type:* 25 – functional seizures  6- Motor symptoms  Rest – any subtype of FND  *Symptom Duration:* Not reported | 49 | IP | *Clinician Type:* pain psychiatry, psychology, counselling, PT, OT, nursing  *Intervention:* Medication management, individual/group psychotherapy, education (type not specified), biofeedback/relaxation techniques, PT, OT. | MDT  Intervention period: 3–4-weeks  Treatment delivered Monday to Friday  *Intensity*: not specified | Subjective measures: Pain-related disability, depression, anxiety and stress scores.  Objective measures: TUAG, SCT, 6MWT.  OT assessment tools not specified.  *Follow-up period:* not specified. | Statistically significant reductions in subjective measures of pain-related disability, depression, anxiety and stress. Significant improvements in objective measures of physical functioning. | OT role not described.  OT intensity not described.  OT assessment tools not specified. |
| Hardin & Carson 2019  (62) | Case report | *Sex: Female*  *Age: 30*  *FND Type:* multiple co-morbidities and mixed functional symptoms.  *Symptom Duration:* Not reported | 1 | IP | *Clinician Type:* physiatrist, psychology, PT, OT.  *Intervention:* Operant behavioural interventions.  Psychotherapeutic interventions; stress management, couples counselling, CBT principles, interpersonal effectiveness training, DBT.  PT and OT – scaffolded treatment plan, gradual reduction of use of aids, reduced attention to symptoms and abnormal movement, behaviours. Praise provided for normal functioning. | MDT  *Intervention period:* 22 days  *Intensity:* not specified | BBS, JHFRAT, walking endurance, use of aids, use of stairs.  OT assessment tools not specified.  *Follow-up period:* not specified. | Discharge: independent transfers, walking good distances. Managing stairs with standby supervision. Improvements in BBS, falls risk reduced. Not using aids.  Frequency of ‘cataplectic’ episodes had reduced.  At follow-up she reported that she had completed a 5km charity walk. | OT role described.  OT intensity not described.  OT assessment tools not specified. |
| Richardson et al 2018.  (35) | Retrospective consecutive case series | *Sex:* 6 males, 6 females  *Age:* 19-63 years, mean 41.2  *FND Type:* 7 – weakness  3- Tremor  2- Functional seizures  2 - Mixed symptoms  *Symptom Duration:* Not reported | 12 | IP (11) and OP (1) | *Clinician Type:* medical staff, clinical psychology, PT, OT, nursing.  *Intervention:*  *Medical staff:* Diagnosis discussion  *Clinical Psychology:* Formulation, psychoeducation, challenge unhelpful beliefs about symptoms.  *PT* – movement re-training.  *OT*- support to re-engage in daily activities.  Graded exposure principles/treatment was incorporated. | MDT  *Intervention period:* Not specified.  *Intensity:*  IPs: 2-4 hours of therapy daily for 5 days.  Nursing support available 24 hrs day  OP: 3 hours total input. | FIM  Video to demonstrate improvement  *Follow-up period:* btw 12- and 26-months post-discharge. Mean follow-up time was 17 months post-discharge.  OT assessment tools not specified | 12/13 = complete or almost complete symptom remission.  Significantly improved functioning compared to admission.  1 patient showed no improvement – did not complete treatment. | OT intensity is not described.  OT assessment tools not specified. |
| Glass et al., 2018.  (56) | Retrospective cohort study | *Sex:* 79 females, 21 men  *Age: mean 39.6*  *FND Type:* functional seizures, functional weakness, Motor symptoms  *Symptom Duration:* 3.8 years (45.6 months). | 100 | OP | *Clinician Type:* neurologist-psychiatrist, PT, OT, psychotherapy.  *Intervention:* Education about the diagnosis,  individualised treatment plans.  PT –followed PT consensus recommendations  For NES – self-help resources + individual psychotherapy.  For mixed symptoms self-help resources were used alongside therapies.  OT role not described. | MDT  *Intensity:* baseline evaluation + up to 6 outpatient visits. | Clinician assessment and patient self-report of clinical improvement, employment or disability status at the time of the last follow-up as compared to baseline.  *Follow-up period:* average of 7 months.  OT assessment tools not specified | 81 patients returned for follow-up: 42% reported some degree of clinical improvements at 7 months.  Treatment adherence was associated with clinical improvement at 7 months.  Having chronic pain was associated with poorer outcomes.  Having functional weakness was reported to be a risk factor for treatment non-adherence. | OT role not described.  OT intensity is not described.  OT assessment tools not specified. |
| Jacob et al., 2018.  (33) | Retrospective cohort study | *Sex:* 24 women, 8 men  *Age: 18 and over,* mean 49.1 years  *FND Type:* Motor symptoms  *Symptom Duration:* Mean symptom duration 7.4 years (88.8 months) | 32 | IP | *Clinician Type:* PT, OT, psychotherapy, SLT, neurology, physiatry, social work.  *Intervention:* Daily PT, OT, psychotherapy and SLT (if required).  Relearning normal movement control through a stepwise approach.  OT role not specifically described.  Physical therapies described as; retraining normal quality, movement. Breathing/relaxation strategies are used. Positive gains verbally re-enforced, and abnormal movements ignored. Assistive devices removed as able. Mental practice re-training. | MDT  *Intervention period:* 1 week  *Intensity:*  3 hours per day of PT, OT, SLT + 1 hour of psychotherapy | CGI, PMDRS, CGI-SR, SDS, HRQoL, Health care visits, BDI, STAI, PC-PTSD-5, SPAQ, Linear Analogue Self-assessment of well-being, BVS, ERQ, SHAI.  Standardised videos of patients taken on first and last day of treatment by PT: scored by movement disorders specialist using the PMDRS.  *Follow-up period:* 6 months.  OT assessment tools not specified | At discharge 86.7% patients reported symptom improvement on the CGI, improvement maintained at 6 months in 69.2%.  PMDRS scores improved by 59.1% from baseline to discharge.  Symptom severity had improved.  Reduction in disability in the social life domain of the SDS but not in work and family life domains.  Physical visits and ED visits reduced from admission to 6-month follow-up.  Reduction in movement disorder symptoms, independence with mobility increased and wheelchair use decreased.  OT was rated as being beneficial on a 10-point Likert scale (9.2/10). | OT role not specifically described.  OT assessment tools not specified. |
| Dahlhauser & Theuer 2017  (20) | Retrospective cohort study | *Sex:* 26 women, 10 men  *Age:* 18-75 (mean 46)  *FND Type:* Motor symptoms  *Symptom Duration:* Not reported | 36 | OP | *Clinician Type:* physical medicine and rehabilitation physician, neurology, OT, PT.  *Intervention:* individualised to patient goals. Common tasks; split attention tasks on a balance ball, walking with a progressive weight in a laundry basket or fine Motor games. Positive re-enforcement of normalised movement, ignoring / distracting from maladaptive movement. Fine Motor and gross Motor skills, diaphragmatic breathing, PMR, good body mechanics within function. | MDT  *Intervention period:* 1 week  *Intensity:*  Ranged from 2 to 10 OT sessions (mean =2)  Up to 2, 45-minute sessions of both OT and PT daily for one week (up to 10 sessions of OT) | COPM  *Follow-up period*: not specified. | Performance scores increased by 3.4 points  Satisfaction by 4.7 points  (Clinically meaningful change – 2 or more). | OT role, intensity and assessment tools are described. |
| Yam and Rickards 2016  (55) | Case report | *Sex: Male*  *Age: 45*  *FND Type:* Mixed functional symptoms  *Symptom Duration:* 1 month | 1 | IP | *Clinician Type:* physiatry, neuro and rehab psychology, OT, PT, SLT, recreational therapy.  *Intervention:* Psychoeducation.  OT: addressed left UL dexterity, co-ordination, shoulder function, balance, co-ordination, ability to bend and squat, attention and communication. Graded exposure, IADL training including cooking, budgeting, path finding, laundry, return to driving. Engaged in program to train service dogs. Community re-integration – volunteering, biofeedback for anxiety, relaxation techniques. | MDT  *Intervention period:* 3 months  Psychotherapy delivered 3 times weekly.  Cognitive rehab groups 3 times weekly  *Intensity* of physical therapies (PT, SLT, OT) not described | BAI, WHOQOL-BREF, MPAI, NSI, PHQ-9, PAI, PLC-5, SWLS.  *Follow-up period:* 6 months. | Three months after admission: improvement in functional symptoms, four months after admission, symptoms had largely resolved. 6 months post-discharge; maintained his gains and was symptom free. Lived independently and independent with IADLs. Full-time job. | OT role is described.  OT intensity not described.  OT assessment tools not specified. |
| Demartini et al., 2014  (54) | Prospective cohort study | *Sex:* 46 women, 20 men  *Age:* Median 43.7  *FND Type:* Motor symptoms (50.5%), functional seizures (21.2%), weakness (18.8%), sensory symptoms (9.5%).  *Symptom Duration:* 4.8 years (57.6 months) | 66 | IP | *Type of clinician:* CBT, OT, PT, neuropsychiatry, neurology.  *Intervention:* Advised referring to Saifee et al for details (same setting). Treatment plan individualised and tailored to patient goals. Combined cognitive and physical rehabilitation; emphasis on developing coping strategies and changing illness beliefs, distraction techniques and error-based learning techniques.  OT role not specifically described. | MDT  *Intervention period:* 4 weeks  *Intensity:*  Therapy delivered Monday – Friday. Intensity not described  OT intensity not specified | HONOS, HADS, PHQ-15, IPQ-R, the Common Neurological Symptom Questionnaire, FQ, COPM.  At discharge and 1-year follow-up, patients also completed 5-point self-rated scales of improvement. 1 year follow-up, patients were sent self-report questionnaire, and a psychiatrist conducted a telephone assessment using a semi-structured interview.  *Follow-up period:* 1 year | Significant improvements in clinician-rated mental health and functional ability.  Mood and anxiety had improved, and patients were less bothered by their somatic symptoms.  2/3 of patients rated their general health as better or much better at discharge –maintained at 12 months.  HONOS scores continued to improve over the following 12 months. | OT role – brief description in Saifee et al., and use of COPM as outcome measure.  OT intensity not described. |
| McCormack et al., 2014  (38) | Retrospective cohort study | *Sex:* 26 women, 7 men  *Age:* mean age of 40.8 years  *FND Type:* Motor symptoms  *Symptom Duration:* median 48 months | 33 | IP | *Clinician Type:* neuropsychiatry, psychology, PT, OT, SLT as required  *Intervention:* Psychoeducation.  Supported graded steps with positive re-enforcement. Approaches adapted to the relevant disability.  OT: assess baseline ADLs and safety, integrate new Motor skills in daily tasks. Encouraged to engage in activities that will test his / her existing skill set. OT assesses perspective on their role and routine e.g.; do they view themselves as disabled. Assessment of home and social environments and impact on function. Re-integration into daily activities and roles.  All professionals developed a relapse prevention plan; identify triggers for symptoms, enhance self-monitoring of behaviours, emotions, symptoms and apply learnt symptom management techniques. | MDT  *Intervention period:* Median 101 days  *Intensity:*  PT – twice weekly  OT intensity not specified | Mobility and ADLs were ranked on an ordinal scale on admission and discharge.  Mobility: clinician rated: walking unaided, walking with aids, wheelchair or bedbound  ADLs: rated as largely independent, somewhat dependent, mostly / fully dependent (clinician rated).  MRS: admission and discharge: 0=no symptoms, 5 = severe disability (clinician rated).  Outcomes were defined as ‘improvement in ADLs from admission to discharge or independent at discharge (yes/no), improvement in mobility from admission to discharge or walking unaided at discharge (yes / no) + LOS (above / below median)  Standardised OT assessment tools not specified.  *Follow-up period*: not specified | 72.2% had improved mobility  87.9% had improved ADLs  72.7% had improved MRS  Mobilising unaided or with the use of a stick / crutches rose from 33 to 72.7%.  Wheelchair dependence fell from 57.6 to 15.2 %  Largely independent with ADLs rose from 57.6% to 78.8%  Ability to be cared for at home rose from 90.9% to 100%.  Having NES + MOTOR correlated with increase LOS. | OT role described.  OT intensity not specified.  Standardised OT assessment tools not used. |
| Jordbru et al., 2014  (31) | Randomised study with waiting-list control. Cross-over design: Patients randomly consecutively and equally (1:1 ratio) allocated to immediate 3 weeks of treatment or 4 weeks on a waiting list. | *Sex:* 48 women and 12 men  *Age:* mean age of 38 years  *FND Type:* Motor symptoms  *Symptom Duration:* mean 10 months | 60 | IP | *Clinician Type:* physician, PT, OT, nurse, educator in adapted physical activity.  *Intervention:* Adaptive physical activity within a cognitive behavioural framework, explanation of symptoms, positively re-enforcing normal gait, not re-enforcing dysfunction.  Daily adapted sports activities (riding a bicycle, ball activities, outdoor canoeing, indoor climbing). Patients were helped to shift their focus from disability to mastering of activities.  Training sessions were undertaken by nursing staff in the evenings.  OT role not specifically described. | MDT  *Intervention period:* 3-week IP admission.  *Intensity:* not specified  OT intensity not specified | Outcomes measured at baseline, admission, discharge, 1-month, and 1-year follow-up using the FIM (clinician rated), FMS (physician rated) and SF-12 (self-reported).  OT assessment tools not specified  *Follow-up period:* 1 year. | Strong and significant carry-over effects on the FMS and FIM.  No patients required a wheelchair or crutches at discharge; majority returned to work and/or independent living.  Patients kept their gains at 1-year follow-up, the only exception being the SF-q12 mental subscale which did not show significant change. | OT role not specifically described.  OT assessment tools not specified.  OT intensity not specified. |
| Czarnecki et al., 2012  (32) | Retrospective cohort study with historical control | *Sex:* 46 women and 24 men  *Age: Btw* 17 -79 (mean 45)  *FND Type:* Motor symptoms  *Symptom Duration:* median 17 months. | 60 – treatment and control (treatment as usual) | OP | *Clinician Type:* OT, PT, SLT, neurology, physiatry, psychiatry, psychology.  *Intervention:* Education about the diagnosis.  Motor-reprogramming to establish normal movement patterns, ignoring abnormal movements using a stepwise strategy. Distraction techniques to extinguish abnormal movements. Positive gains verbally re-enforced, abnormal movements ignored. Strategy repetition encouraged.  OT role not specifically described. | MDT  *Intervention period:* Mean 4.7 / 5 days  *Intensity:*  Twice daily PT and OT for 5 consecutive days + SLT if required | Primary outcome: improvement of the movement disorder (patient and physician rated GGI-I) + long-term outcome (patient-rated).  Secondary outcome: self-rated level of disability in daily activities.  Video was used to demonstrate change to patients.  OT assessment tools not specified.  *Follow-up period:* median 25 months. | Discharge: 69% of patients in treatment group improved, nearly normal or in remission (clinician -rated).  Follow-up: Treatment group: 60.4% markedly improved, almost completely normal or in remission. 25% were no better than mildly improved.  Control group; 21.9% were markedly improved, almost completely normal / in remission. | OT role not specifically described.  OT assessment tools not specified but have measured self-rated level of disability. |
| Saifee et al., 2012  (53) | Retrospective cohort study | *Sex:* 15 women and 11 men  *Age:* mean age of 47  *FND Type:* motor symptoms; 68%, weakness; 16% and fatigue 16%.  *Symptom Duration:* mean 3 years (36 months). | 26 | IP | *Clinician Type:* neuro PT, neuro OT, CBT, nursing, neuropsychiatry, neurology.  *Intervention:* Individualised to patient goals.  Combined cognitive and physical rehabilitation; developing coping strategies and changing illness beliefs, distraction and error-based learning techniques.  OT role not specifically described. | MDT  *Intervention period:* Median LOS: 24 days (15 – 32 days)  Therapy delivered Monday to Friday. Intensity not described  OT intensity not specified | VAS: impact of symptoms on function.  Assess the impact of symptoms and beliefs about symptoms using the ‘cause’ component of the illness perception questionnaire and WSAS.    VAS: benefit of programme.  OT assessment tools not specified.  *Follow-up period:* 2 years | 58% reported benefit from the program on discharge.  Self-reported benefit to symptoms was largely maintained after a 2-year period but RTW and cessation of benefits was uncommon, even in those that had improved.  OT reported by patients as the most beneficial component of MDT treatment. | OT role not specifically described.  OT intensity not described.  OT assessment tools not specified.  OT reported by patients as the most beneficial component of treatment for their FND. |
| Chou et al 2006  (67) | Case report | *Sex: Male*  *Age: 50*  *FND Type:* Functional weakness  *Symptom Duration:* Assessment at onset | 1 | IP | *Clinician Type:* neurology, psychiatry, PT, OT  *Intervention:* behavioural modification program to restore power, function and mood. Psychologic intervention. FES used as an adjunct.  OT role not described | MDT  *Intervention period:* Not specified  OT intensity not specified | Not specified.  OT assessment tools not specified.  *Follow-up period:* Not specified | Significant improvements in muscle power, functional abilities, and ADLs. | OT role not described.  OT intensity not defined.  OT assessment tools not specified. |
| Heruti et al 2002  (68) | Case series | *Sex:* 25 men, 9 women  *Age:* mean age of 30.7  *FND Type:* Functional paralysis  *Symptom Duration:* Not specified | 34 | 2 IP rehab facilities | *Clinician Type:* physiatry, nursing, PT, OT, social work, psychology. Psychiatry available as required.  *Intervention Type:* Described as: based on an inter-disciplinary team approach.  OT role not described | MDT  *Intervention period:* Not fully described for each case.  OT intensity not specified | Not specified.  *Follow-up period:* Not specified | Functionally, 9 patients had a complete recovery, 10, a partial recovery and 15 remained unchanged. | OT role not described.  OT intensity not described.  OT assessment tools not specified. |
| Speed, 1996  (52) | Case series | *Sex:* 5 men, 5 women  *Age:* 19 to 69 (mean; 32.7 years)  *FND Type:* functional weakness.  *Symptom Duration:* 0.5 weeks to 28 months. | 10 | IP | *Clinician Type:* neurology, physiatry, psychiatry, PT, OT, recreation therapy, psychological therapies.  *Intervention:* Behavioural approach  Education about the diagnosis.  Patient confined to wheelchair, except during therapy.  PT: Gait re-training.  Recreation therapy.  Psychological therapies; stress management, family education.  OT: positive re-enforcement of normal function, promoting standing in functional tasks.  *Note:* Treatment protocol published as an appendix. | MDT  *Intervention period:* Mean LOS: 11.8 days  OT intensity not specified | Clinician rated: FIM ambulation score.  Descriptive data: duration of symptoms & treatment.  Symptom recurrence, new neurological symptoms, new treatment, new psychiatric or neurological diagnosis post-discharge.  OT assessment tools not specified.  *Follow-up period*: *2 - 27 months.* | Normal ambulation attained at discharge.  At follow-up, 7 of 9 patients maintained normal ambulation. 2 patients resumed partial dependence on wheelchair use.  No patient had symptom substitution or new diagnosis in the follow-up period. | OT role described.  OT intensity not described.  OT assessment tools not specified. |
| Vatine et al., 1996 (61) | Case series | *Sex:* 2 men  *Age:* 40 yrs (Case 1); 18 yrs (Case 2)  *FND Type:* Functional weakness (Case 1); functional paralysis (case 2)  *Symptom Duration:* Approx 2 months (Case 1); 6 weeks (Case 2) | 2 | IP | *Clinician Type:* rehabilitation physician, psychiatry psychologist, PT, OT  *Intervention:* Education about the diagnosis. Behavioural rehabilitation approach with PT, OT and psychotherapy. Positive reinforcement of normal function graded functional rehabilitation.  OT: Gradual introduction of the upper limb into functional tasks. Incorporated positive reinforcement of normal movement, coordination, pinch and dexterity exercises within functional activities to facilitate recovery of upper limb function. | MDT  *Intervention period:* approx. 4-6 weeks  Psychotherapy twice weekly and psychiatric review weekly. Frequency and duration of physical therapies (OT + PT) not specified. | Non-standardised clinical assessment. Follow-up evaluation of symptom recurrence and functional recovery.  OT assessment tools not specified.  Follow-up period: Case 1: 1 month and 6 months; Case 2: 6 months | Case 1: Gradual recovery of upper limb movement, at 6 weeks only slight limitation of active lifting; symptom free at 4-week follow-up and remained symptom free at 6m.  Case 2: demonstrated progressive improvement during admission. On discharge full function. Returned to work (military training). Symptom free at 6-month follow-up. | OT role described  OT intensity not described  OT assessment tools not specified. |
| Paget & Rigby, 1996  (42) | Case report | *Sex:* Female  *Age:* 38  *FND Type:* TBI, spinal injuries and functional overlay.  *Symptom duration:* 10 days post injury at start of treatment. | 1 | CNRT | *Clinician Type:* OT, SLT, PT. Psychological therapies offered but declined.  *Intervention:*  Patients’ identified goals of; learning to read, improving concentration, memory, returning to work, becoming more socially active. Full description not provided. | MDT  *Intervention period:* 10 days post injury for a 4-month period + 9 months post injury (after self-referral) for a 3-month period. Discharged at 12 months post-injury.  OT intensity not specified | Clinician rated: AMPS, RBMT, COTNAB  *Follow-up period:* not specified. | Improved functional independence with daily activities, improved written and verbal language skills, cognitive abilities.  Remained dependent on benefits and care. | Specific descriptions of OT intervention and intensity not described.  OT assessment tools specified.  Despite functional improvements, patient continued to receive benefits and care. |
| McDade & Brown 1992  (41) | Case series | *Sex:* 9 males, 7 females  *Age:* mean age of 34.1  *FND Type:* Functional seizures and dual diagnosis of functional seizures and epilepsy  *Symptom duration:* average of 168 months), mean 10.5 months. | 16 | IP | *Clinician Type:* psychiatry, psychotherapy, OT, nursing, art therapy, music therapy, PT if required, family therapy if required.  *Intervention:* Education about the diagnosis.  Psychotherapy, OT and operant  Conditioning model. Medication optimisation. Functional seizures managed by staff by directing minimal attention to them.  PT - re-training for gait disorders.  OT role not described | MDT  *Intervention period:* 12 weeks to 6 months  OT intensity not specified | Seizure frequency.  OT assessment tools not specified.  *Follow-up period:* 1 year. | 8: seizure free at the end of treatment, 3: occasional functional seizures, 5: unchanged.  Improvements in social functioning and reduced use of healthcare resources.  Results stable at 1-year follow-up. | OT role not described.  OT intensity not specified.  OT assessment tools not specified. |
| Fishbain et al 1988  (37) | Case series | *Sex:* 3 males, 1 woman  *Age:* 41, 47, 77, 60 (mean 56)  *FND Type*: functional paralysis  *Symptom duration*: 42 to 156 months (mean 79.5 months). | 4 | IP | *Clinician Type:* neurosurgery, PT, OT, behavioural modification for pain, vocational counselling and FES. One patient: psychotherapy.  Intervention: Education about the diagnosis.  EMG feedback technique to eliminate belief that the affected muscle was paralysed and to improve strength of the affected muscle.  OT role not described | MDT  *Intervention period:* 2 weeks to 3.5 months.  EMG – ranging from 70 sessions over 3.5-month period, 40 sessions over 1 month, 7 sessions over 2 weeks  OT intensity not specified | Clinician rated.  Increase in strength measured via isometric maximum voluntary contract and EMG.  Use of mobility aids, walking tolerance, number of step-ups, standing tolerance, number of clutch presses.  OT assessment tools not specified.  *Follow-up period:* 4 months to 4 years. | Significantly improved levels of function at discharge, reduction in use of aids. Symptoms had resolved at follow-up. | OT role not described.  OT intensity not specified.  OT assessment tools not specified. |
| Withrington & Parry, 1985  (36) | Case series | *Sex:* 5 women  *Age:* 33, 27, 33, 30, 42 (mean 33)  *FND Type:* functional weakness.  *Symptom duration:* 12 years to 168 months, mean 58.4 months) | 5 | IP | *Clinician Type:* doctor, PT, OT, clinical psychologist, social worker, nursing.  *Intervention:* Education about the diagnosis, Motor retraining, and biofeedback.  OT role not described | MDT  *Intervention period:* 4 weeks to 6 months.  OT intensity not specified | Muscle charting  Video recording  *Follow-up period:* 3 months to 3 years.  OT assessment tools not specified. | All demonstrated significant improvements in symptoms and function on discharge and follow-up. | OT role not defined.    OT intensity not specified.  OT assessment tools not specified. |

#### **Key:**

*Treatment Type:* IP – In-patient, OP – Out-patient, CNRT – Community Neuro-Rehabilitation Team, MDT - Multidisciplinary

*Symptom Type*: MOTOR – Functional Movement Disorder, FNS – Functional Neurological Symptoms

*Clinicians:* OT – occupational therapy, PT – Physiotherapy, SALT – Speech and Language Therapy

*Outcome Measures:* **AMPS** – Assessment of Motor and Process Skills, **RBMT** - The Rivermead Behavioural Memory Test, **COTNAB** - The Chessington OT Neurological Assessment Battery, **FIM** – Functional Independence Measure, **VAS** – Visual Analogue Scale, **WSAS** – Work and Social Adjustment Scale, **FMS** – Functional Mobility Scale, **SF-12**- Health related quality of life scale, **MRS** – Modified Rankin Scale, **HONOS** – Health Of Nation Outcome Scale, **HADS** – Hospital Anxiety and Depression Scale, **PHQ-15** – Patient Health Questionnaire somatic symptoms subscale, **PHQ-9** – Patient Health Questionnaire depression scale, **QPR** – Process of Recovery Questionnaire, **SWEMWBS** – Short Warwick Edinburgh Well-being Scale, **GAD7** – Generalised Anxiety Disorder 7-point scale, **RSE** - Rosenberg Self-Esteem scale, **SPIN** - Social phobia inventory, **EQ-5D-5L** – Health status questionnaire, **DSSS** – Depression and Somatic Symptoms Scale, **PAI-BOR** – Personalised Assessment for Borderline Personality Disorder, **RFL** – Reasons for Living Scale, **KIMS** – The Kentucky Inventory of Mindfulness Skills, **PAI** - Personality Assessment Inventory, **BVS** - Body Vigilance Scale, **BBS** – Berg Balance Scale, **JHFRAT** - John Hopkins Falls Risk Assessment Tool, **TUG** - Timed up and go, **SCT** - Stair climbing test, **6MWT** - 6-minute walk test, **ERQ** - Emotional Response Questionnaire, **SHAI** - The Short Health Anxiety Inventory, **PCL-5** - PTSD Checklist, **STAI** - The State-Trait Anxiety Inventory, **CDS –** Clinical Decision Support, **Euro-QoL** - measure of health-related quality of life , **IPQ-R** – Illness Perception Questionnaire, **PC-PTSD-5** - Primary Care PTSD Screen, **SPAQ** - Sexual and Physical Abuse Screen Questionnaire, **SWLS** - Satisfaction with Life Scale, **IPQ-R** - Revised Illness Perception Questionnaire, **FQ** - The Fear Questionnaire, **COPM** – Canadian Occupational Performance Measure, **BAI** - Beck Anxiety Inventory, **BDI** – Becks Depression Inventory, **WHOQOL-BREF** – Quality of Life assessment, **MPAI** - Mayo Portland Adaptability Inventory, **NSI** - Neurobehavioural Symptom Inventory, **CGI-S** – Clinical Global Impression Scale - Severity of Illness, **CGI-I** - Clinical Global Impression Scale – Global Improvement, **EQVAS** – Global assessment of health visual analogue scale, **PMDRS** – Psychogenic Movement Disorders Rating Scale, **AASP** – Adolescent Adult Sensory Processing Tool, **PSFS** - Patient specific functional scale, **BBT** - Box and Block Test, **DGI** – The Dynamic Gait Index, **SDS** – Sheehan Disability Scale, **S-MOTORRS** – Simplified Functional Movement Disorders Rating Scale, **HRQoL** – Health-related Quality of Life, **CORE** – Clinical Outcomes in Routine Evaluation, **DERS** – Difficulties in Emotional Regulation Scale, **PLC-5** – PTSD Checklist, **GROC** – Global Rating Of Change, **MAS** – Motor Assessment Scale, **FGA** – Functional Gait Assessment, **TUG** – Timed Up and Go, **FIST** – Function In Sitting Test, **IRF-PAI** - IP Rehabilitation Facility Patient Assessment Instrument, **MoCA** – Montreal Cognitive Assessment, **IUS-12** – Intolerance of Uncertainty, **MiniBestest**, **Sensory Organisation Test**, **PII** – Postural Instability Index, **DHI** – Dizziness Handicap Inventory.

## 4. Table 2: Detailed study characteristics and intervention outcomes (Occupational Therapy)

| ***Ref*** | ***Study Design*** | ***Primary FND presentation*** | ***n*** | ***Setting*** | ***Intervention & Intensity*** | ***OT Assessment Tools*** | ***Outcomes*** |
| --- | --- | --- | --- | --- | --- | --- | --- |
| Foong et al., 2026 (63) | Retrospective cohort study | Mixed symptoms + predominant pain | 100 | IP | MDT  *Intervention period:* 4 weeks  *OT intensity:* Not specified. MDT treatment delivered across 4 weeks.  *OT intervention:* Not described  OT intensity not described. | COPM  Follow-up period: Not specified | COPM (n=83): Performance +2.8–3.5; Satisfaction +4.2–4.5 (clinically meaningful in both groups; NS between groups) |
| Rush et al., 2025 (46) | Prospective cohort study | Motor symptoms | 45 | OP | MDT  *Intervention period:* 5 days  *OT intensity:* 3-4 1 hour sessions over 5 days  *OT intervention:* Focused on deconstructing abnormal movement /postures, normal movement re-training.  Relaxation exercises, introduction of cognitive and environmental challenges to promote carry over into real world scenarios. | COPM  Follow-up period: Not specified | COPM: Performance +3.86, Satisfaction +5.20 (clinically meaningful) |
| Le Perf et al., 2025 (66) | Retrospective cohort study | PPPD  Dizziness | 20 | IP | MDT  *Intervention period:* 4 weeks  *OT Intensity:* Not specified – treatment delivered daily, 5 days per week for 4 weeks.  *OT Intervention:* balance group training and functional mobility (gait circuits, static balance tasks); embedded within MDT; focus on functional rehabilitation and postural control | OT assessment tools not specified  *Follow-up period:* Not specified | Significant improvements across balance, mobility, postural control, and disability outcomes. |
| Gilmour et al., 2025 (45) | Case series | Motor symptoms: 100% | 20 | OP | MDT  *Intervention period:* 5 days, embedded within a 12-month pathway.  *OT Intensity:* 1-hr OT delivered on day 2 & 3 of the 5 day programme.  *OT Intervention:* Not fully described; goal-directed task specific rehabilitation. | COPM  *Follow-up period:*  6 weeks and 12 months | COPM (n=20): mean performance: +2.9 [SD 2.1], satisfaction: +3.9 [SD 2.4]  EQ5D5L (n=20): mean + 10.2 [SD 17.6]  Goal achievement (n=15): 93% mostly or entirely agreed.  Involvement in decisions about care (n=15): 100% mostly or entirely agreed. |
| Zheng et al., 2025 (51) | Case report | Functional seizures  Functional Tremor  Functional weakness  Speech disturbance  Cognitive dysfunction | 1 | IP | MDT  *Intervention period:* 8 days  *OT intensity*:daily  *OT intervention:* graded activity practise incorporating psychobehavioural approaches and using CBT informed strategies. | OT assessment tools not specified  *Follow-up period:* Not specified | Improved independence in daily activities (to independent/supervision level), increased upper and lower limb strength, improved static and dynamic balance for falls prevention, and improved cognitive function including short-term memory and problem-solving. Admission MOCA: 22/30, discharge score not specified. |
| Schneider et al., 2024  (50) | Retrospective cohort study | Motor symptoms: 13.3%, dysarthria 6.7%, functional seizures 3.3%, functional hemiparesis 53.3%,  sensory impairment 60%. | 30 | IP | MDT  *Intervention period:* mean 4 weeks.  *OT Intensity:* 1 daily session of OT for 30-45 minutes, 5 days per week.  *OT Intervention:*  Specific therapy roles not defined. | FIM  Work status at admission and follow-up.  OT assessment tools not specified.  *Follow-up period:*  mean: 36 months. | Good in 22/30 at discharge with a degree of functional independence for all Motor FIM items and in 21 at follow-up.  56.7% working at discharge and 40% working at follow-up.  Disability insurance payouts: 43.3 % prior to admission and 60% at follow-up.  10% experienced relapse requiring a second hospitalisation; one after 25 months, one after 27 months, and another at 38 months. |
| Saunders et al., 2024  (40) | Retrospective cohort study | Mixed symptoms; 60%, functional seizures; 17%, Motor symptoms; 14%, cognitive symptoms; 6%, extreme fatigue;4%. | 52 | IP | MDT  *Intervention period:* mean 19.4 weeks, range; 3-76 weeks.  *OT Intensity:* not specified.  *OT Intervention:* activity planning, relaxation strategies, building confidence, and life skills. | OT assessment tools not specified  *Follow-up period:* Not specified | 43/52 patients’ global condition had improved on the CGI-I; 12 very much improved, 12 much improved, 19 some improvement, 8 no benefit.  1 patient deteriorated (pre-existing mental health condition).  Measures of mobility, depression, and quality of life significantly improved.  Symptoms were experienced as more understandable and less distressing. |
| Polich et al., 2024  (48) | Retrospective cohort study | Limb weakness 86.4%,  generalised FND 27.3%,  functional paraplegia 18.2%, functional hemiplegia 36.4%, gait disorder 95.5%, speech / swallow/voice 13.6%,  cognitive symptoms 40.9%, functional seizures 22.7%. | 22 | IP | MDT  *Intervention period*: 2 weeks  *OT Intensity:* 3 or more hours of therapy delivered daily over 5 to 6 days per week. Most time spent in OT and PT.  *OT Intervention:*  Aquatic therapy co-delivered by PT and OT.  Therapeutic recreation sessions offered individually and with PT/OT.  OT consensus recommendations for FND applied.  Self-management plan completed.  Patients referred for community therapy on discharge if required. | Physical and OT measures on the IRF-PAI.  *Follow-up period:* not specified. | Statistically significant changes in admission and discharge measures of self-care, transfers, ambulation and balance.  90% of patients showed improvements on the OT summary variable.  95% showed improvements on the PT transfer summary variable.  At discharge, significantly more patients were ambulatory, walking independently and without assistive devices. BBS improved to a statistically significant degree.  1 /22 patients showed no improvement. |
| McCombs et al., 2024  (27) | Retrospective cohort study | Motor symptoms: 57  Functional seizures: 29  Speech: 21. | 77 | OP | OT (11) / MDT (66)  11 patients stand-alone OT,  45 patients’ concurrent psychotherapy, 39 concurrent PT, 17 concurrent SLT.  *Intervention period:* 1-6 months.  *OT Intensity:* 1-hr OT treatment sessions, starting with once weekly, reduction in frequency to once every few weeks.  *OT Intervention:*  8 OT sensory-based treatment modules previously described (10)  Sessions: in person and remote. | AASP  Primary outcome; clinician-rated improvement rating; somewhat improved, much improved.  *Follow-up period:* not specified. | Patients reported sensory processing patterns with elevated scores; low registration, sensory sensitivity, sensory avoidance compared with normative values.  62% of the patients were ‘improved’ post-sensory-based OT intervention. |
| Callister et al 2023  (43) | Retrospective cohort study | Motor symptoms | 201 | OP | MDT  *Intervention period:* 5 days  *OT Intensity:* 3-4 1-hour sessions daily between PT and OT.  *OT Intervention:*  Therapeutic screening offered to some patients.  Motor-retraining in line with PT, OT consensus recommendations for FND.  Relaxation exercises, self-management strategies. Functional task practise. | COPM  GROC  Retrospective investigator-rated scale (1-3 – worse /not improved to significantly improved/resolved).  *Follow-up period*: discharge or within 30 days post-completion. | Substantial improvement in COPM performance and satisfaction scores (mean 3.8 SD 1.9).  GROC (mean 5.5, SD 1.7)  Investigator-rated outcomes – 84% moderately to significantly improved/resolved.  3% of patients who underwent the screening improved and did not require admission to the program. |
| Ashrafzdeh 2023  (65) | Case report | Motor symptoms, functional seizures with Cushing’s Disease. | 1 | IP | MDT  *Intervention period:* not specified.  *OT Intensity:* not specified.  *OT Intervention: e*ducation, OT for hand dexterity: writing, drawing, stress ball. | OT assessment tools not specified  *Follow-up period:* Not specified | Regained hand function (able to eat and write). Able to stand and take steps with assistance. Reduced seizure frequency. Improvements in speech quality. Mental health, functional symptoms improved. |
| Geary 2022  (49) | Case series | Motor symptoms, functional weakness, dizziness, functional seizures. | 3 | IP | MDT  *Intervention period:* 13 to 18 days  *OT Intensity:* 3 hours of therapy per week between PT, OT.  *OT Intervention:* Education re: FND.  OT role not described. | OT assessment tools not specified  *Follow-up period:*  CS 1 and 3 not specified. CS 2: ‘a few months after discharge’ | Each patient made functional gains by discharge: improved transfers, gait, ability to manage stairs, reduced supervision/assistance required. |
| Hebb et al 2022  (60) | Case report | Motor symptoms | 1 | IP | MDT  *Intervention period:* 8 days  *OT Intensity:* not described  *OT Intervention:* not specifically described. | OT assessment tools not specified  *Follow-up period:* 4 years | Overall improvements in gait with resolution of compensatory movements.  4-year follow-up – gait disorder remained resolved, but fatigue and pain limited walking endurance. |
| Reid et al., 2022.  (44) | Case series | Motor symptoms | 18 | OP | MDT  *Intervention period:* 5 days.  *OT Intensity:* 90 minutes daily.  *OT Intervention:* Autonomic regulation and Motor re-training. Diaphragmatic breathing, vagal nerve stimulation, mindfulness re-training. Motor re-training within functional tasks, whilst incorporating mental imagery. | COPM  *Follow-up period:* not specified. | Significantly improved Motor and gait outcomes.  Improved patient perception of functional performance |
| MacLean et al., 2022  (25) | Case studies | Mixed FNS | 2 | OP | MDT  Case study 1; received PT alongside OT. OT *Intensity:* OT once every 2 weeks for 8 weeks (7 sessions).  Case study 2: received CBT, SLT alongside OT.  *OT Intensity:* OT once weekly for 8 weeks (7 sessions).  *OT Intervention:* physical examination, observation of Motor skills, sensory processing assessments (AASP), review of daily routine and impact of symptoms on function. Individualised sensory-based treatment plan. | AASP  COPM  Case Study 1: follow-up period: 4 & 8 months  Case Study 2: follow-up period not specified. | Case study 1: Increased self-awareness of sensory preferences, independent use of sensory strategies. Improved Motor control, stress management.  COPM scores improved: performance by 3.6, satisfaction by 5.  FNS ‘much improved’ at follow-up.  Case study 2:  Reduced seizure frequency, stress and anxiety, increased community access, improved cognition.  COPM: performance increased by 3.4, satisfaction by 2.2. |
| Schmidt et al 2021  (59) | Prospective cohort study | Motor symptoms | 31 | IP | MDT  *Intervention period:* Range: 10-35 days, median; 21 days.  *OT Intensity:* not specified.  *OT Intervention:* not specifically described. | OT assessment tools not described  *Follow-up period*: average 5 months, range 1-20 months. | Significantly reduced functional movement symptom severity at discharge and follow-up. |
| Hebert et al., 2021.  (58) | Case series | Motor symptoms | 17 | IP | MDT  *Intervention period:* median 7.5 days (range 3-14 days).  *OT Intensity:* not described.  *OT Intervention:* Physical therapies: Motor re-training, identification of symptom triggers, maintaining factors.  OT role not specifically described. | Physical therapies assessment tools: MAS, FGA, BBS, TUG, FIST, FIM Motor scale.  OT specific assessment tools not specified  *Follow-up period:* 12 months (13/17 completed follow-up) | Improvements in function, depression, anxiety, and somatic symptoms at discharge.  1-year follow-up: minimal sustained improvement and worsening psychopathology. |
| Henry et al 2021  (39) | Case report | Functional seizures and functional weakness | 1 | IP | MDT  *Intervention period:* 12-months.  *Intensity:* Intensity of physical therapies not described.  *Intervention:* OT role not described. | OT assessment tools not specified  *Follow-up period*: Weekly follow-ups with care co-ordinator for 6 months. | Improvements on all outcome measures at discharge and 6-month follow-up.  On discharge, patient was self-harm free, walking unaided and no longer met EUPD criteria.  Reduction in use of mobility aids.  Seizure free at discharge. |
| Joos and Halmer 2020  (57) | Case report | Motor symptoms | 1 | IP | MDT  *Intervention period:* 8 weeks  *OT Intensity:* not specified.  *OT Intervention:* Integrated IP rehabilitation, including mirror therapy.  OT used manual therapy to relax muscles alongside mirror therapy. | OT assessment tools not specified  *Follow-up period:* 2 months | Tremor substantially decreased after 8 weeks. Improved FMC, improved hand-eye co-ordination.  Improvement sustained at 2-month follow-up. |
| Faul et al 2020  (34) | Case series | Motor symptoms | 14 | IP | MDT  *Intervention period:* 5 days  *OT Intensity:* 3 hours daily physical therapy between PT, OT, SLT (if necessary)  *OT Intervention:*  Emotional go / No Go fMRI task before and after MoRE treatment: Motor re-training. | OT assessment tools not specified  *Follow-up period:* Not reported | PMDRS scores significantly reduced after treatment. Significant improvement in symptoms. |
| Petrochilos et al., 2020  (47) | Case series | 50% predominant Motor symptoms, 41% functional seizures, 9% sensory or cognitive symptoms, 81% mixed symptoms. | 78 | OP | MDT  *Intervention period:* 5 weeks  *OT Intensity:* 2 sessions of OT per week for 5 weeks + MDT review session.  Sessions: up to 1 hour.  *OT Intervention:*  Group FND education.  Re-engagement with activities. Aim to reduce reliance on equipment and care. Self-management principles, graded goal setting. Symptom management strategies. Fatigue, pain, anxiety management, improving structure and routine, graded activity practise, community access, assisting with return to vocational roles.  Relapse prevention planning.  Discharge family meeting  6-month review | COPM. Collected at admission, discharge, 6-month review.  *Follow-up period:* 6 months. | Statistically significant improvements in somatic symptoms as measured on the PHQ15, depression (PHQ9), anxiety (GAD7), health and social functioning (HONOS), function (COPM), health status (EQ-5D-5L) and patient rated clinical global improvement (CGI) (discharge and 6-month review). |
| Jimenez et al 2019  (64) | Case series | 25 – Functional seizures  6- Motor symptoms  Rest – any subtype of FND | 49 | IP | MDT  *Intervention period:* 3–4-weeks. Treatment delivered Monday to Friday.  *OT Intensity:* not specified.  *OT Intervention:* education, biofeedback/relaxation techniques, OT detail not specified. | OT assessment tools not specified  *Follow-up period:* not specified | Statistically significant reductions in subjective measures of pain-related disability, depression, anxiety and stress. Significant improvements in objective measures of physical functioning. |
| Hardin & Carson 2019  (62) | Case report | Multiple co-morbidities and mixed functional symptoms. | 1 | IP | MDT  *Intervention period:* 22 days  *OT Intensity*: not specified.  *OT Intervention:* PT and OT – scaffolded treatment plan, Motor-retraining, gradual reduction of use of aids. | OT assessment tools not specified  *Follow-up period:* not specified | Discharge: independent transfers, improved walking endurance (up to 5km). Managing stairs with supervision. Improvements in balance, falls risk reduced. Not using aids.  Frequency of ‘cataplectic’ episodes had reduced. |
| Richardson et al 2018.  (35) | Case series | *FND Type:*  7 – weakness  3- Tremor  2- Functional seizures  2 - Mixed symptoms  *Symptom Duration:* Not reported | 12 | IP (11) & OP (1) | MDT  *Intervention period and intensity:* IPs: 2-4 hours of therapy daily for 5 days.  OP: 3 hours total input.  *OT Intervention:* support re-engagement in daily activities. Graded exposure. | FIM  Video to demonstrate improvement  OT assessment tools not specified  *Follow-up period:* btw 12- and 26-months, mean -17 months post-discharge. | 12/13 = complete or almost complete symptom remission. Significantly improved functioning compared to admission.  1 patient showed no improvement – did not complete treatment. |
| Glass et al., 2018.  (56) | Retrospective cohort study | Functional seizures, functional weakness, motor symptoms | 100 | OP | MDT  *Therapy intensity:* baseline evaluation + up to 6 out-patient visits.  *OT Intervention:*  Education  OT role not described. | Clinician assessment and patient self-report of clinical improvement, employment or disability status at the time of the last follow-up as compared to baseline.  OT assessment tools not specified  *Follow-up period:* average of 7 months. | 81 patients returned for follow-up: 42% reported some degree of clinical improvement at 7 months. |
| Jacob et al., 2018.  (33) | Retrospective cohort study | Motor symptoms | 32 | IP | MDT  *Intervention period:* 1 week  *OT Intensity:* 3 hours per day of physical therapies: PT, OT, SLT  *OT Intervention:* OT role not explicitly described. Physical therapies: Motor re-training, relaxation strategies. Assistive devices removed as able. Mental practice re-training. | OT assessment tools not specified  *Follow-up period:* 6 months | At discharge, 86.7% reported symptom improvement on CGI, improvement maintained at 6 months in 69.2%. PMDRS scores improved by 59.1% from baseline to discharge.  Symptom severity had improved.  Reduction in disability in the social life domain of the SDS but not in work and family life domains.  Physical and ED visits reduced from admission to 6-month follow-up.  Improved FMS, independence, mobility, reduced wheelchair use.  OT rated as being beneficial on a 10-point Likert scale (9.2/10). |
| Dahlhauser & Theuer 2017  (20) | Retrospective cohort study | Motor symptoms | 36 | OP | MDT  *Intervention period:* 1 week  *OT Intensity:* up to 2, 45-minute sessions of both OT and PT daily for one week (up to 10 sessions of OT).  *OT Intervention:* individualised to patient goals. Common tasks; split attention tasks on a balance ball, walking with a progressive weight in a laundry basket or fine Motor games. Relaxation techniques. | COPM  *Follow-up period:* not specified. | Performance scores increased by 3.4 points  Satisfaction by 4.7 points  (Clinically meaningful change – 2 or more). |
| Yam and Rickards 2016  (55) | Case report | Mixed functional symptoms | 1 | IP | *MDT*  *Intervention period:* 3 months  *OT Intensity:* not described.  *OT Intervention:* Psychoeducation. UL rehab, balance, co-ordination, ability to bend and squat, attention and communication. Graded exposure, IADL training. Anxiety, relaxation techniques. Community reintegration. | OT assessment tools not specified.  *Follow-up period:* 6 months. | Three months after admission: improvement in functional symptoms. Four months after admission: symptoms had largely resolved. Six months post-discharge: maintained his gains and was symptom free. Lived independently and independent with IADLs. Full-time job. |
| Demartini et al., 2014  (54) | Prospective cohort study | Motor symptoms (50.5%), functional seizures (21.2%), weakness (18.8%), sensory symptoms (9.5%). | 66 | IP | MDT  *Intervention period:* 4 weeks  Therapy delivered Monday – Friday.  *OT Intensity*: not described.  *OT Intervention:* Combined cognitive and physical rehabilitation.  OT role not specifically described. | COPM  5-point self-rated scales of improvement.  *Follow-up period:* 1 year | Significant improvements in clinician-rated mental health and functional ability.  Mood and anxiety improved, and patients were less bothered by their somatic symptoms.  2/3 of patients rated their general health as better or much better at discharge –maintained at 12 months.  HONOS scores continued to improve over the following 12 months. |
| McCormack et al., 2014  (38) | Retrospective cohort study | Motor symptoms | 33 | IP | MDT  *Intervention period*: median 101 days  *OT Intensity:* OT intensity not specified  *OT Intervention:* Psychoeducation. Graded steps with positive re-enforcement. Assess baseline ADLs and safety, integrate Motor re-training in daily tasks. Assessment of role and routine, home and social environments and impact on function.  All professionals: relapse prevention plan. | Mobility and ADLs: ordinal scale on admission and discharge.  Mobility: clinician rated: walking unaided, walking with aids, wheelchair or bedbound. ADLs: largely independent, somewhat dependent, mostly / fully dependent (clinician rated).  MRS: admission and discharge: 0=no symptoms, 5 = severe disability (clinician rated).  Outcomes were defined as ‘improvement in ADLs or independent (yes/no), improvement in mobility or walking unaided (yes / no) + LOS (above/below median)  Standardised OT assessment tools not specified.  *Follow-up period*: not specified. | 72.2% had improved mobility, 87.9% had improved ADLs, 72.7% had improved MRS.  Mobilising unaided or with stick/crutches rose from 33 to 72.7%.  Wheelchair dependence fell from 57.6 to 15.2 %.  Largely independent with ADLs rose from 57.6% to 78.8%.  Ability to be cared for at home rose from 90.9% to 100%. |
| Jordbru et al., 2014  (31) | Randomised study with waiting-list control | Motor symptoms | 60 | IP | MDT  *Intervention period:* 3 weeks.  *OT Intensity*: Not specified.  *OT Intervention:* Adaptive physical activity within a CBT framework. OT role not specifically described. | Outcomes measured at baseline, admission, discharge, 1-month, and 1-year follow-up using the FIM (clinician-rated), FMS (physician-rated) and SF-12 (self-reported).  OT assessment tools not specified  *Follow-up period:* 1 year. | Strong and significant carry-over effects on FMS, FIM.  No patients required a wheelchair or crutches at discharge, majority RTW and/or independent living.  Maintained at 1-year follow-up, exception being the SF-q12 mental subscale: no significant change. |
| Czarnecki et al., 2012  (32) | Retrospective cohort study with historical control | Motor symptoms | 60 | OP | MDT  *Intervention period:* mean 4.7 / 5 days.  *OT Intensity:* Twice daily PT, OT for 5 consecutive days  *OT Intervention:* Education. Motor-reprogramming. OT role not specifically described. | Primary outcome: improvement of MOTOR (patient and physician rated GGI-I) + long-term outcome (patient-rated).  Secondary outcome: self-rated level of disability in daily activities.  Video to demonstrate change.  OT assessment tools not specified  *Follow-up period:* median 25 months. | Discharge: 69% of treated patients improved, nearly normal or in remission (clinician-rated).  Follow-up: Treatment group: 60.4% markedly improved, almost completely normal or in remission. 25% were no better than mildly improved. Control group; 21.9% markedly improved, almost completely normal / in remission. |
| Saifee et al., 2012  (53) | Retrospective cohort study | Movement disorders; 68%, weakness; 16%, fatigue 16%. | 26 | IP | MDT  *Intervention period:* Median LOS: 24 days (15 – 32 days)  *OT Intensity:* Monday-Friday. Intensity not described.  *OT Intervention:*  Combined cognitive and physical rehabilitation. OT role not specifically described. | VAS: impact of symptoms on function.  WSAS.  VAS: benefit of programme.  OT assessment tools not specified.  *Follow-up period:* 2 years | 58% reported benefit from the program on discharge.  Self-reported benefit to symptoms was largely maintained after 2 years, but RTW, cessation of benefits was uncommon.  OT reported by patients: most beneficial component of MDT treatment. |
| Chou et al 2006  (67) | Case report | Functional weakness | 1 | IP | MDT  *Intervention period:* Not specified  *OT intensity:* not specified.  *OT Intervention:* not specified. | OT assessment tools not specified  *Follow-up period:* Not specified | Significant improvements in muscle power, function, ADLs. |
| Heruti et al 2002  (68) | Case series | Functional paralysis | 34 | 2 IP rehab facilities | MDT  *Intervention period:* Not fully described for each case.  *OT intensity:* not specified.  *OT intervention:* not described | OT assessment tools not specified  *Follow-up period:* Not specified | 9 patients had complete recovery, 10 partial recovery, 15 remained unchanged. |
| Speed, 1996  (52) | Case series | Functional weakness. | 10 | IP | MDT  *Intervention period:* Mean LOS: 11.8 days.  OT intensity: not specified  *OT Intervention:* positive re-enforcement of normal function, promoting standing in functional tasks. | Clinician rated: FIM ambulation score.  Duration of symptoms & treatment. Symptom recurrence, new neurological symptoms, new treatment, new psychiatric or neurological diagnosis post-discharge.  OT assessment tools not specified  *Follow-up period:* 2 -27 months. | Normal ambulation at discharge.  At follow-up, 7 of 9 patients maintained normal ambulation, 2 patients resumed partial dependence on wheelchair use.  No patient had symptom substitution or new diagnosis in the follow-up period. |
| Vatine et al., 1996 (61) | Case series | Functional weakness (Case 1); functional paralysis (case 2) | 2 | IP | MDT  *Intervention period:* 4-6 weeks  *OT intensity:* not specified  *OT intervention:* Gradual introduction of the upper limb into functional tasks. Incorporated positive reinforcement of normal movement, coordination, pinch and dexterity exercises within functional activities to facilitate recovery of upper limb function. | OT assessment tools not specified  Follow-up period: Case 1: 1 and 6 months; Case 2: 6 months | Case 1: Gradual recovery of upper limb movement, at 6 weeks only slight limitation of active lifting; symptom free at 4-week follow-up and remained symptom free at 6m.  Case 2: demonstrated progressive improvement during admission. On discharge full function. Returned to work (military training). Symptom free at 6-month follow-up. |
| Paget & Rigby, 1996  (42) | Case report | TBI, spinal injuries and functional overlay. | 1 | CNRT | MDT  *Intervention period:* 10 days post injury for 4 months + 9 months post injury (after self-referral) for 3 months. Discharged at 12 months.  *OT intensity*: Not specified  *OT Intervention:* Cognitive rehab, RTW goals, social activities. Full OT description and intensity not provided. | Clinician rated: AMPS, RBMT, COTNAB  *Follow-up period:* not specified. | Improved: functional independence, written and verbal language skills, cognitive abilities.  Remained dependent on benefits and care. |
| McDade & Brown 1992  (41) | Case series | Functional seizures + dual diagnosis of functional seizures and epilepsy | 16 | IP | MDT  *Intervention period:* 12 weeks to 6 months.  *OT intensity:* not described  *OT Intervention:* not described | OT assessment tools not specified  *Follow-up period:* 1 year | 8: seizure free at discharge, 3: occasional NES, 5: unchanged.  Improvements in social functioning, reduced healthcare.  Results stable at 1-year follow-up. |
| Fishbain et al 1988  (37) | Case series | Functional paralysis | 4 | IP | MDT  *Intervention period:* 2 weeks to 3.5 months.  *OT intensity:* not described  *OT Intervention:* Education, EMG feedback. | Increase in strength measured via isometric maximum voluntary contract and EMG.  Use of mobility aids, walking tolerance, number of step-ups, standing tolerance, number of clutch presses.  OT assessment tools not specified.  *Follow-up period:* 4 months to 4 years. | Significantly improved function at discharge, reduction in use of aids. Symptoms had resolved at follow-up. |
| Withrington & Parry, 1985  (36) | Case series | Functional weakness. | 5 | IP | MDT  *Intervention period:* 4 weeks to 6 months.  *OT intensity:* not described.  *OT Intervention:* not described | OT assessment tools not specified  *Follow-up period:* 3 months to 3 years | All: significant improvements in symptoms and function on discharge and follow-up. |

**Key:**

*Treatment Type:* IP – In-patient, OP – Out-patient, CNRT – Community Neuro Rehabilitation Team, MDT - Multidisciplinary

*Symptom Type:* MOTOR – Functional movement disorder, FNS – Functional neurological symptoms

*OT Outcome Measures:* **AMPS** – Assessment of Motor and Process Skills, **RBMT** - The Rivermead Behavioural Memory Test, **COTNAB** - The Chessington OT Neurological Assessment Battery, **FIM** – Functional Independence Measure, **VAS** – Visual Analogue Scale, **WSAS** – Work and Social Adjustment Scale, **FMS** – Functional Mobility Scale, **SF-12**- Health related quality of life scale, **MRS** – Modified Rankin Scale, **HONOS** – Health Of Nation Outcome Scale, **Euro-QoL** - measure of health-related quality of life , **COPM** – Canadian Occupational Performance Measure, **AASP** – Adolescent Adult Sensory Processing Tool, **GROC** – Global Rating Of Change, **MAS** – Motor Assessment Scale, **IRF-PAI** - IP Rehabilitation Facility Patient Assessment Instrument

### Table 3. Commonly used standardised outcome measures used in rehabilitation for FND

| **Occupational Therapy Specific Outcome Measures** | | | |
| --- | --- | --- | --- |
| **Outcome measure** | **Administration**  **(patient or clinician-rated)** | **Health domain** | **Studies using the outcome measure** |
| Canadian Occupational Performance Measure (COPM) (74) | Clinician | Occupational performance | 8 (20, 25, 43, 45-47, 54, 63) |
| Adult Adolescent Sensory Processing Tool (AASP) (84) | Clinician | Symptom severity (sensory)  Occupational performance | 2 (25, 27) |
| Assessment of Motor and Process Skills (AMPS) (91) | Clinician | Occupational performance | 1 (42) |
| Chessington OT Neurological Assessment Battery (COTNAB) (85) | Clinician | Occupational performance | 1 (42) |
| **Commonly Used Outcome Measures in Multidisciplinary Rehabilitation** | | | |
| Clinical Global Scale of Improvement (CGI-I) (CGI-S) ^a^ (94) | Patient or clinician | Symptom severity | 6 (33, 34, 40, 47, 58, 59) |
| Psychogenic Movement Disorders Rating Scale (PMDRS) (79)  Simplified Functional Movement Disorders Rating Scale (S-FMDRS) ^a^ (87) | Clinician | Symptom severity | 3 (33, 34, 59)  1 (59) |
| Berg Balance Scale (BBS) (98) | Clinician | Physical functioning (balance) | 6 (40, 44, 49, 58, 60-62) |
| 10-metre walk test (10MWT) (81) | Clinician | Physical functioning (walking speed) | 3 (40, 49, 60) |
| Timed Up and Go (TUAG) (83) | Clinician | Physical functioning (walking speed) | 3 (40, 44, 64) |
| Functional Independence Measure (FIM) (93) | Clinician | Functional ability (daily activities) | 5 31, 35, 50, 52, 58) |
| Work and Social Adjustment Scale (WSAS) ^a^ (96) | Patient | Functional ability (daily activities) | 2 (47, 53) |
| Generalised Anxiety Disorder (GAD-7) (89) | Patient | Psychological health (anxiety) | 4 (39, 47, 58, 63) |
| Stait-Trait Anxiety Inventory (STAI) (88) | Patient | Psychological health (anxiety) | 3 (33, 34, 40) |
| Beck Anxiety Inventory (BAI) ^a^ (90) | Patient | Psychological health (anxiety) | 1 (55) |
| Hospital Anxiety and Depression Scale (HADS) ^a^ (97) | Patient | Psychological health (anxiety and depression) | 1 (54) |
| Patient Health Questionnaire (PHQ-9) (92) | Patient | Psychological health (depression) | 4 (39, 55, 58, 63) |
| Health of the Nation Outcome Scores (HoNOS) (95) | Clinician | Psychological health (health and social functioning) | 2 (47, 54) |
| EuroQol 5-Dimension 5-Level questionnaire (EQ-5D-5L) (80) | Patient | Quality of Life (health related) | 4 (40, 45, 47, 63) |
| EuroQol Group’s quality of life instrument (Euro-QoL) (82) | Patient | Quality of Life (health related) | 1 (40) |
| Health-Related Quality of Life (HRQoL) (86) | Patient | Quality of Life (health related) | 1 (33) |

a= Outcome measures recommended for FND research in a systematic review and consensus statement (49).
